# A Targeted *In Vivo* RNAi Screen Reveals Deubiquitinases as New Regulators of Notch Signaling

**DOI:** 10.1534/g3.112.003780

**Published:** 2012-12-01

**Authors:** Junzheng Zhang, Min Liu, Ying Su, Juan Du, Alan Jian Zhu

**Affiliations:** Department of Cell Biology, Lerner Research Institute, Cleveland Clinic, Cleveland, Ohio, 44195

**Keywords:** deubiquitinase, *Drosophila melanogaster*, Notch signaling, ubiquitination

## Abstract

Notch signaling is highly conserved in all metazoan animals and plays critical roles in cell fate specification, cell proliferation, apoptosis, and stem cell maintenance. Although core components of the Notch signaling cascade have been identified, many gaps in the understanding of the Notch signaling pathway remain to be filled. One form of posttranslational regulation, which is controlled by the ubiquitin-proteasome system, is known to modulate Notch signaling. The ubiquitination pathway is a highly coordinated process in which the ubiquitin moiety is either conjugated to or removed from target proteins by opposing E3 ubiquitin ligases and deubiquitinases (DUBs). Several E3 ubiquitin ligases have been implicated in ubiquitin conjugation to the receptors and the ligands of the Notch signaling cascade. In contrast, little is known about a direct role of DUBs in Notch signaling *in vivo*. Here, we report an *in vivo* RNA interference screen in *Drosophila melanogaster* targeting all 45 DUBs that we annotated in the fly genome. We show that at least four DUBs function specifically in the formation of the fly wing margin and/or the specification of the scutellar sensory organ precursors, two processes that are strictly dependent on the balanced Notch signaling activity. Furthermore, we provide genetic evidence suggesting that these DUBs are necessary to positively modulate Notch signaling activity. Our study reveals a conserved molecular mechanism by which protein deubiquitination process contributes to the complex posttranslational regulation of Notch signaling *in vivo*.

Notch signaling is an evolutionarily conserved developmental pathway that is strictly required to direct the specification of almost every cell type (Artavanis-Tsakonas *et al.* 1999; Lai 2004). Furthermore, Notch signaling functions to regulate stem cell maintenance and adult tissue homeostasis (Liu *et al.* 2010). Given the wide-ranging importance of Notch signaling in development, it is not surprising that dysregulation of Notch signaling in humans results in birth defects as well as tumor formation in different organs (Bolós *et al.* 2007; Ranganathan *et al.* 2011; Louvi and Artavanis-Tsakonas 2012; Penton *et al.* 2012; South *et al.* 2012).

First identified in *Drosophila*, the *Notch* gene encodes a type I transmembrane receptor protein with two known ligands, Delta and Serrate, which themselves are type I transmembrane proteins. Binding of the Notch receptor with its ligands expressed by the neighboring cell initiates the signaling cascade, leading to a sequence of proteolytic events that releases the Notch intracellular domain (NICD). The NICD subsequently translocates into the nucleus, where it associates with Suppressor of Hairless [Su(H)] and Mastermind (Mam) proteins to assemble an active transcription complex that selectively turns on the expression of downstream target genes (Fortini 2009; Kopan and Ilagan 2009).

Notch signaling is tightly controlled both in time and space, and such complex regulation can occur at multiple levels. For example, the regulation of the amount of Notch receptors and respective ligands, proteolytic processing to generate active NICD, the formation of transcriptional repressive or active complexes on the chromatin, as well as trafficking of both receptors and ligands have all been shown as crucial steps for modulating Notch signaling output (Schweisguth 2004; Bray 2006; Fortini 2009; Andersson *et al.* 2011).

Protein ubiquitination represents a major form of posttranslational regulatory mechanism that modulates protein quality control, stability and trafficking (Grabbe *et al.* 2011). The ubiquitination process is catalyzed by three distinct enzyme complexes, which conjugate small modifier protein ubiquitin (Ub) to specific substrates in a step-wise fashion. A single E1 (Ub-activating enzyme) and a single E2 (Ub-conjugating enzyme) complex are responsible for activating and conjugating Ub moiety, respectively. In contrast to a limited number of E1 and E2 enzymes, distinct E3 complexes serve as the Ub protein ligases to transfer Ub moiety from the E2 enzyme onto specific substrates. A fourth type of enzyme, the deubiquitinating enzyme (also known as deubiquitinase or DUB), counteracts the ubiquitination process by removing Ub from substrate proteins (Ciechanover 2005; Grabbe *et al.* 2011).

During the past decade, genetic screens performed in *Drosophila* and other model organisms, combined with rigorous biochemistry, have begun to unveil the importance of ubiquitination in the regulation of Notch activity. Several E3 ubiquitin ligases have been identified to regulate ubiquitination of the Notch receptor (Lai 2002; LeBras *et al.* 2011; Weinmaster and Fischer 2011). Suppressor of deltex [Su(dx)] was first discovered in *Drosophila* as a negative regulator of Notch signaling, acting in an antagonist manner to another E3 ubiquitin ligase Deltex identified in later studies (Matsuno *et al.* 1995; Cornell *et al.* 1999; Qiu *et al.* 2000; Matsuno *et al.* 2002). Along with the other two E3 ligases, Nedd4 and c-Cbl, Su(dx) plays important roles in sorting and lysosomal degradation of unactivated Notch receptor (Jehn *et al.* 2002; Sakata *et al.* 2004; Wilkin *et al.* 2004; Wang *et al.* 2010). An evolutionarily conserved F-box protein Fbw7 (also known as Sel-10) has been shown to ubiquitinate NICD, resulting in proteasomal degradation of NICD in *Caenorhabditis elegans* and mammals, although a direct demonstration of its role in *Drosophila* is missing (Hubbard *et al.* 1997; Oberg *et al.* 2001; Wu *et al.* 2001; Tetzlaff *et al.* 2004; Tsunematsu *et al.* 2004; Matsumoto *et al.* 2011; Nicholson *et al.* 2011). In addition, work from several laboratories indicated that two distinct E3 ligases, Neuralized (Neur) and Mind bomb (Mib1), directly promote mono-ubiquitination of the ligand proteins Delta and Serrate to facilitate their endocytosis (Deblandre *et al.* 2001; Lai *et al.* 2001; Pavlopoulos *et al.* 2001; Yeh *et al.* 2001; Itoh *et al.* 2003; Chen and Corliss 2004; Lai *et al.* 2005; Le Borgne *et al.* 2005). Furthermore, clonal analysis in *Drosophila* suggested that *neur* and *mib1* function in the signal-sending cell and are required for most Notch-mediated processes (Le Borgne and Schweisguth 2003; Li and Baker 2004; Pitsouli and Delidakis 2005; Wang and Struhl 2005).

On the contrary, very little is known about the role of DUBs in the regulation of Notch signaling. To date, the only DUB enzyme that has been characterized in Notch signaling in *Drosophila* is Fat facets (Faf), a ubiquitin carboxyl-terminal hydrolase domain-containing protein that shares homology with vertebrate USP9 (Chen and Fischer 2000). Studies in the fly eye development revealed that Faf enhances Delta endocytosis to promote Notch signaling to ensure a correct recruitment of photoreceptor precursors (Cadavid *et al.* 2000; Overstreet *et al.* 2004). In this context, Faf deubiquitinates Liquid facets (Lqf), the *Drosophila* Epsin homolog, to increase the activity of Lqf, which in turn enhances the efficiency of Delta internalization (Chen *et al.* 2002). Very recently, the eukaryotic translation initiation factor 3 complex subunit F (eIF3F) was identified as a DUB to positively regulate Notch signaling in cultured mammalian cells (Moretti *et al.* 2010), but whether it functions in the same manner *in vivo* is unknown. In addition, three DUBs have been implicated as potential regulators of Notch signaling in a genome-wide screen for zebrafish development (Tse *et al.* 2009). However, whether they function directly on the Notch signaling pathway is undetermined.

The patterning of the adult fly wing blade represents a simple but efficient *in vivo* system for studying Notch signaling (Blair 2007). Here, we describe an *in vivo* RNA interference (RNAi) screen targeting all 45 DUBs that we annotated in the fly genome (Supporting Information, Table S1) to identify DUBs as novel modulators of Notch signaling. By monitoring the effects of reduced expression of individual DUBs on the formation of the wing marginal vein and the specification of scutellar macrochaetae (bristles), we identified at least four DUBs as potential Notch signaling modulators. Our genetic analyses provided evidence demonstrating that *CG8445* (*calypso*), *CG9124* (*eIF-3p40*), and *CG9769*, which encode the *Drosophila* orthologs of vertebrate BAP1 and the eIF3 complex subunits H and F, respectively, play a conserved role in regulating Notch signaling *in vivo*. In addition, we characterized *CG32479*, the *Drosophila* ortholog of vertebrate USP10, whose activity is necessary and sufficient to positively regulate Notch signaling.

## Materials and Methods

### Fly genetics

*Act5C*-Gal4, *Act5C*>*yw* > Gal4, *C96*-Gal4, *dpp*-Gal4, *GMR*-Gal4, *ptc*-Gal4, *tub*-Gal4, *Gbe*+*Su(H)m8-lacZ* [*Su(H)-lacZ*], and *neuralized^A101^-lacZ* (*neur-lacZ*) were described previously (Huang *et al.* 1991; Furriols and Bray 2001; Ahmed *et al.* 2003; Kim *et al.* 2006; Du *et al.* 2011; Su *et al.* 2011). Transgenic RNAi flies targeting all 45 DUB genes predicted in the fly genome were obtained from the Vienna *Drosophila* RNAi Center [VDRC, Vienna, Austria (Dietzl *et al.* 2007)], and the Fly Stocks of National Institute of Genetics (NIG-Fly; National Institute of Genetics, Shizuoka, Japan; Table S2). Our primary RNAi screens were conducted at 29° by crossing individual RNAi lines with the *dpp*-Gal4 and *c96*-Gal4 drivers, respectively, for defects on patterning the adult wing margin and scutellar bristles. Crosses were shifted to lower temperatures (18° or 21°) for RNAi lines whose overexpression resulted in embryonic or larval lethality at 29° (Du *et al.* 2011).

We took three approaches to ensure the specificity and effectiveness of DUB RNAi transgenes used in our targeted screens. (1) In most cases, we obtained at least two RNAi lines targeting different regions of a specific DUB, thus avoiding potential off-target effects associated with a particular dsRNA construct. (2) For those DUBs with only a single RNAi line available, we obtained transgenic GS (Gene Search) trap fly lines (Toba *et al.* 1999) from the *Drosophila* Genetic Resource Center (Kyoto, Japan) to test effects of overexpressed DUB. One example using this approach was *CG32479*, in which overexpression or downregulation of *CG32479* in the scutellum resulted in opposite phenotypes on the differentiation of scutellar bristles (Figure 8). (3) For those DUBs with a single RNAi line whose RNAi knockdown did not cause any obvious defects in the wing or the scutellum, we searched databases of existing genome-wide RNAi screens for the effectiveness of these RNAi lines when overexpressed by other Gal4 drivers in other cellular processes (Cronin *et al.* 2009; Mummery-Widmer *et al.* 2009; Pospisilik *et al.* 2010; Neely *et al.* 2010a,b; Saj *et al.* 2010; Neumüller *et al.* 2011; Valakh *et al.* 2012). For example, a single RNAi line for *CG8830* had no effect in our screen (Table S2). However, the same RNAi line, when overexpressed by the *pnr*-Gal4 driver, resulted in lethality during pupal development (Mummery-Widmer *et al.* 2009).

In a secondary screen to establish a direct involvement of candidate DUBs in the regulation of Notch signaling, expression patterns of Notch signaling targets [Cut, Wingless (Wg) and/or *Su(H)-lacZ*] as well as different forms of the Notch receptor protein [Notch extracellular domain (NECD) and Notch intracellular domain (NICD)] were examined in early third-instar larval wing discs in which DUB RNAi was induced by the *dpp*-Gal4 or *ptc*-Gal4. In addition, cell-autonomous effects of *CG9769* or *CG32479* on Notch signaling were examined in FLIPout clones overexpressing respective RNAi using the *Act5C*>*yw* > Gal4 recombined with UAS-*gfp*, which allow the distinction between RNAi-overexpressing (positively marked by GFP) and wild-type control cells (GFP-negative) in the developing wing (Ito *et al.* 1997). The conditions to induce FLIPout clones in the wing disc are listed as follows: 2-day-old larval progenies from the cross of *hs-flp*;; *Act5C > yw>*Gal4 and UAS-*CG9769* RNAi (V101465; VDRC); UAS-*P35* or UAS-*CG32479* RNAi (V37858 or V37859) were heat-shocked at 37° for 30 min (Su *et al.* 2011). Heat-shocked larvae were then cultured either at 25° (for *CG9769* RNAi) or at 18° (for *CG32479* RNAi) until the third-instar larval stage.

For RNAi lines displaying scutellar bristle defects, *ptc*-Gal4; *neur-lacZ* flies were used to examine the effect of DUBs in the specification of scutellar sensory organ precursor (SOP) cells in the notal region of the wing disc. In some experiments, *Notch* RNAi lines (V27228 and V27229; VDRC), *gfp* RNAi lines (9330 and 9331; Bloomington *Drosophila* Stock Center) and GS lines inserted *in cis* at the *CG32479* locus [202182 and 204397; *Drosophila* Genetic Resource Center at Kyoto, (DGRC), Japan] were used.

### Immunofluorescence of wing imaginal discs and image acquisition of adult fly structures

Wing discs dissected from third-instar larvae were fixed in 4% paraformaldehyde and labeled with the following primary antibodies: mouse anti-Cut [1;100; 2B10; Developmental Studies Hybridoma Bank (DSHB)], mouse anti-Hindsight (Hnt; 1:50; 1G9; DSHB), mouse anti-Wg (1:200; 4D4; DSHB), mouse anti-NECD (1:200; C458.2H; DSHB), mouse anti-NICD (1:200; C17.9C6; DSHB), and rabbit anti-β-Galactosidase (1:4000; Cappel). Alexa fluor-conjugated secondary antibodies (1:400; Invitrogen) were used. In some experiments, DAPI (0.05 μg/mL; Sigma-Aldrich) was used to visualize nuclei. The fluorescence images were acquired with a Zeiss Axio Imager2 microscope equipped with an ApoTome.

Adult eyes, wings, and nota were dissected and mounted as described previously (Zhu *et al.* 2003). The images of these adult structures were acquired with a Leica DMIL inverted microscope (wings) or a Leica MZ16F stereomicroscope equipped with a QImaging QICAM Fast 1394 digital camera (eyes and nota). The figures were assembled in Adobe Photoshop CS5. Minor image adjustments (brightness and/or contrast) were done in AxioVision 4.8.1 or Adobe Photoshop.

## Results

### The genomic inventory of DUBs encoded in *Drosophila melanogaster*

DUBs are highly conserved proteases that catalyze the cleavage of the isopeptide bond between the C-terminal glycine of ubiquitin or ubiquitin-like protein and a unique lysine of a target protein. Recent bioinformatic studies in yeast and vertebrates (Buus *et al.* 2009; Sowa *et al.* 2009; Tse *et al.* 2009; Kouranti *et al.* 2010) further classified DUBs into five subfamilies on the basis of their distinct signature DUB catalytic domains: ubiquitin C-terminal hydrolases (UCHs), ubiquitin-specific proteases (USPs), Machado-Joseph disease domain proteases (MJDs), otubain proteases (OTUs) and JAB1/MPN/Mov34 domain proteases (JAMMs). Among them, the UCHs, USPs, MJDs, and OTUs are cysteine proteases whereas the JAMMs are metalloproteases (Nijman *et al.* 2005).

A handful of DUBs have been studied, and their function has been linked to various cellular processes, including ubiquitin processing, histone modification, cell-cycle regulation, and developmental signaling (Nijman *et al.* 2005; Reyes-Turcu *et al.* 2009; Sowa *et al.* 2009). More importantly, mutations in several DUBs in humans have been identified in diseases, such as cancer, neurodegenerative diseases, and inflammatory diseases (Nijman *et al.* 2005; Singhal *et al.* 2008; Hussain *et al.* 2009; Huang *et al.* 2011), suggesting that the activity of DUBs must be tightly regulated in order to maintain the necessary levels of ubiquitination. Despite the apparent importance of DUBs in many physiological and pathological processes, molecular functions of the majority of DUBs remain largely unknown.

**Figure 1:**
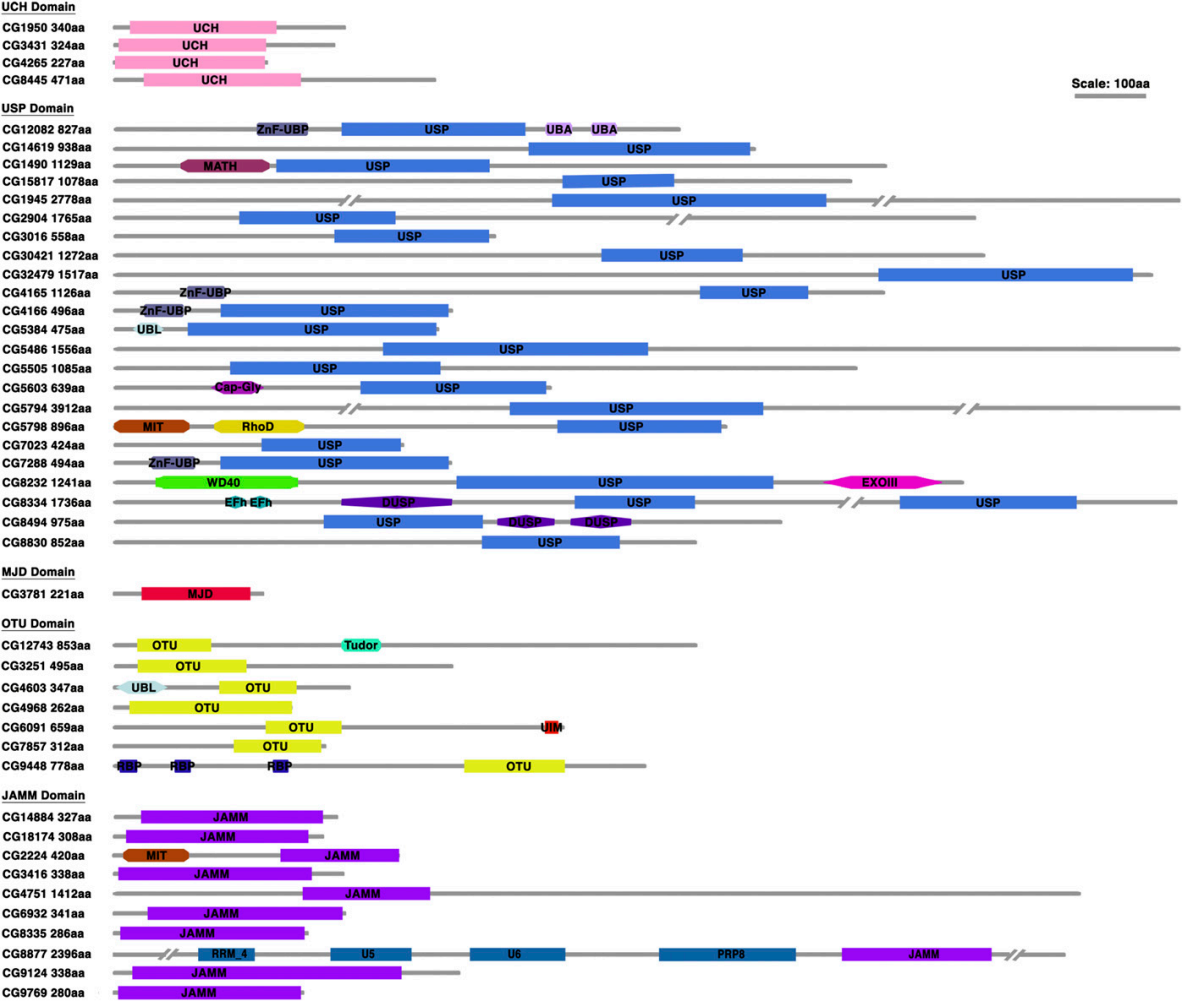
Inventory and domain architectures of annotated DUBs in *Drosophila*. *Drosophila* DUBs are characterized into five subfamilies on the basis of their signature DUB catalytic domains. The UCH, USP, MJD, OUT, and JAMM domain-containing proteases are shown. Apart from signature DUB domains, we retrieved domain architectures for each DUB by using the Pfam and the NCBI Conserved Domain database. The abbreviations for additional domains are listed as follows: Cap-Gly, cytoskeleton-associated proteins, glycine-rich domain; DUSP, domain in ubiquitin-specific proteases; EFh, EF-hand, calcium binding motif; EXOIII, exonuclease, RNase T/DNA polymerase III; MATH, meprin and TRAF-homology; MIT, microtubule interacting and trafficking molecule domain; PRP8, pre-mRNA processing splicing factor 8; RBP, zinc finger, RanBP2-type; RhoD, rhodanese homology domain; RPT, internal repeats; RRM_4, RNA recognition motif of the spliceosomal PrP8; Tudor, Tudor domain; U5 and U6, U5 and U6 snRNA binding domains; UBA, ubiquitin-associated; UBL, ubiquitin-like; UIM, ubiquitin interaction motif; WD40, WD40-repeat-containing domain; and ZnF-UBP, zinc finger ubiquitin binding domain. Proteins and domains are plotted on an approximate scale.

The *Drosophila melanogaster* represents an ideal model system for systematically studying *in vivo* functions of DUBs in many cellular processes, due to its genomic and functional conservation with vertebrates and a large collection of already existing genetic resources. However, the annotation of the fly DUBs is incomplete (Chen and Fischer 2000). We used several bioinformatic tools [Interpro Protein Sequence Analysis (http://www.ebi.ac.uk/interpro/), NCBI Conserved Domain database (http://www.ncbi.nlm.nih.gov/cdd/), and Pfam 26.0 (http://pfam.sanger.ac.uk/)] and found that the fly genome encodes a set of proteins with distinct signature DUB catalytic domains (Figure 1). A total of 45 putative DUBs belonging to five typical DUB sub-families were identified. These included four UCHs, 23 USPs, one MJD proteases, seven OTU proteases, and 10 JAMM proteases (Table S1). Among the 45 fly DUBs identified, 43 were found to have direct orthologs in vertebrates as revealed by NCBI HomoloGene (http://www.ncbi.nlm.nih.gov/homologene) or OrthoDB (http://cegg.unige.ch/orthodb5). The only exceptions are two Otu domain-containing proteases, including the founding member of the Otu sub-family, Ovarian cancer [Otu; CG12743 (Makarova *et al.* 2000)].

DUBs have been demonstrated to play essential roles in multiple cellular processes other than ubiquitin processing. Additional architecture domains that are present in the primary amino acid sequence of these enzymes may provide hints to distinct functions of DUBs. Therefore, we performed domain search by using the Pfam and the NCBI Conserved Domain database, which revealed a wide range of functionally important domains in the fly DUBs (Figure 1). One example is the ZnF-UBP domain (zinc-finger ubiquitin binding domain), which is present at the N-terminal to the USP domain in several fly DUBs, including CG12082, CG4165, CG4166 (Nonstop, Not), and CG7288. CG4166 and its orthologs (yeast Ubp8 and vertebrate USP22; Figure S1) function as an essential component of the SAGA complex to deubiquitinate histone H2B (Henry *et al.* 2003; Weake *et al.* 2008; Zhang *et al.* 2008; Zhao *et al.* 2008). Crystal structure analyses revealed that the Ubp8 ZnF-UBP domain does not form the ubiquitin tail-binding pocket as found in USP5 (vertebrate ortholog of CG12082) and USP16. Instead, it functions as the scaffold to facilitate the assembly of the SAGA complex, thus providing structural insight as to why CG4166 and its orthologs alone do not possess the DUB activity (Reyes-Turcu *et al.* 2006; Allen and Bycroft 2007; Pai *et al.* 2007; Köhler *et al.* 2010; Samara *et al.* 2010). We believe that the conservation of functionally important architecture domains present in the fly DUBs, such as the ZnF-UBP domain, established guidelines for detailed structure/function analyses of specific roles of the fly DUBs in a variety of cellular processes.

### A targeted *in vivo* RNAi screen to identify novel DUB genes in the regulation of Notch signaling

Most of the components of the Notch signaling pathway were identified through classical forward genetic screens conducted in *Drosophila* (Artavanis-Tsakonas *et al.* 1999; Bray 2006; Andersson *et al.* 2011). However, these screens failed to uncover a role of DUBs in the regulation of Notch signaling, probably attributable to the fact that many DUBs exhibit pleiotropic effects in early development. The only exception is *faf*, which encodes a DUB that regulates Notch signaling specifically in the developing eye (Fischer-Vize *et al.* 1992). Recently, the availability of two collections of transgenic RNAi libraries housed in the VDRC (Dietzl *et al.* 2007) and the NIG-Fly Stock Center made it possible to conduct reverse genetic screens to identify DUBs that regulate Notch signaling at later stages of *Drosophila* development. Notch signaling is critical for patterning the wing margin and scutellar bristle (de Celis *et al.* 1991; Artavanis-Tsakonas *et al.* 1999; Brennan *et al.* 1999). We therefore examined the adult wing margin morphology and counted the number of scutellar bristles in the scutellum as simple readouts in our primary *in vivo* RNAi screens to identify DUB genes that function in Notch signaling.

**Figure 2:**
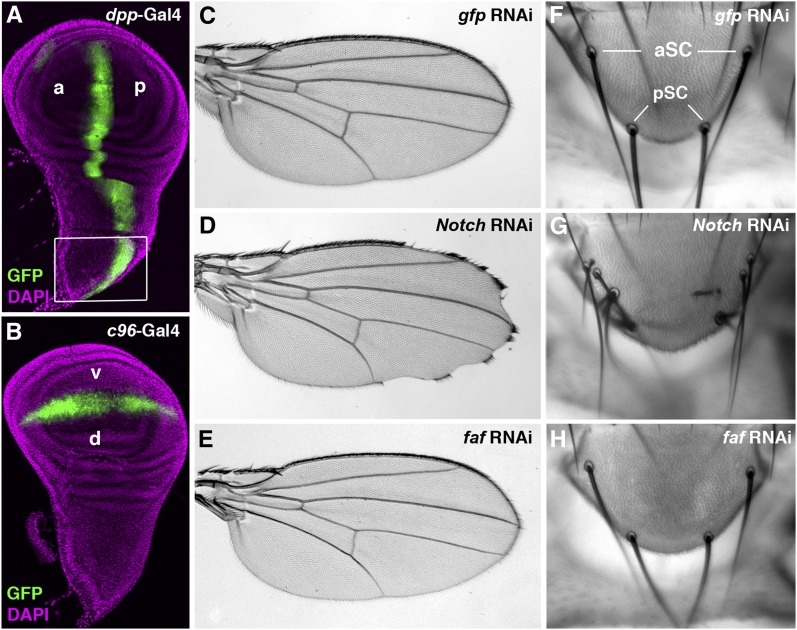
The adult wing margin and scutellar bristle phenotypes are simple but effective readouts for altered Notch signaling. The larval wing imaginal disc is the primordial tissue of the adult wing blade and notum. The expression patterns of the *dpp*-Gal4 (A) and the *C96*-Gal4 drivers (B) in wing discs were marked by the UAS-*gfp* transgene (green). The *dpp*-Gal4 drives transgene expression abutting the anterior-posterior (a-p) boundary (A), whereas the *C96*-Gal4 confers *gfp* expression along the dorsal-ventral (d-v) boundary, *i.e.*, presumptive wing margin in the wing pouch (B). Note that the *dpp*-Gal4 is also expressed in regions where the adult scutellar structures are derived (box; A). DAPI staining was used to mark nuclei in wing discs (magenta; A and B). As expected, ectopic expression of *gfp* RNAi by the *C96*-Gal4 did not produce any effect in the adult wing margin (C). In contrast, reduced expression of *Notch* receptor gene by RNAi resulted in serrations along the wing margin (D). However, knockdown of the expression of *faf*, which encodes a DUB that specifically regulates Notch signaling in the developing *Drosophila* eye, had no effect on patterning of the wing margin (E). Note that wings dissected from female flies are shown in all figures. Two pairs of scutellar bristles, anterior SC and posterior SC (aSC and pSC), are present in the scutellum (F). When the expression of *Notch* was knocked down by RNAi using the *dpp*-Gal4, an increased number of scutellar bristles was observed (G). In contrast, overexpressing RNAi targeting either *gfp* (F) or *faf* (H) had no effect on the specification of scutellar bristles. Phenotypes shown in panels D (n > 20) and G (n = 10) are fully penetrant.

We first reduced the expression of individual DUB genes in wing discs by RNAi using the *dpp*-Gal4 and *C96*-Gal4 drivers, respectively, and then examined the resulting phenotypes in the adult fly. The *dpp*-Gal4 driver confers the RNAi knockdown in the region between longitudinal veins L3 and L4 in the adult wing as well as in the notum (Figure 2A), potentially affecting both the wing margin formation and scutellar bristle differentiation. However, strong *dpp*-Gal4 activity is also observed in embryonic segmentation (Staehling-Hampton *et al*. 1994), a stage that is essential for embryogenesis. In contrast, the *C96*-Gal4 driver dictates the RNAi expression along the wing margin (Figure 2B), thus minimizing a potential effect of embryonic lethality resulted from RNAi overexpression by the *dpp*-Gal4 driver. Note that the *C96*-Gal4 driver also confers a restricted expression in late embryos (Kim *et al.* 2006).

To test whether the aforementioned strategy was feasible for our screens, we examined the effect of RNAi specifically targeting two known regulators of Notch signaling on the wing margin formation and scutellar bristle differentiation. The *Notch* receptor gene is ubiquitously expressed. Flies with reduced *Notch* expression often exhibit scalloped wing margin morphology and defects in the notum (de Celis *et al.* 1991; Artavanis-Tsakonas *et al.* 1999; Brennan *et al.* 1999). Similarly, knocking down *Notch* expression by RNAi using the *C96*-Gal4 driver led to frequent serrations along the wing margin (Figure 2D). Furthermore, we found that *Notch* RNAi, when overexpressed in the notum by the *dpp*-Gal4 driver, led to formation of more scutellular bristles (Figure 2G). *faf*, on the other hand, is an eye-specific regulator of Notch signaling; overexpressed *faf* RNAi by the *tub*-Gal4 driver led to a rough eye appearance (Figure S4B). However, no obvious morphologic defect was observed in the wing (Figure 2E) or scutellum (Figure 2H) when *faf* RNAi was misexpressed in the wing disc by the *C96*-Gal4 or *dpp*-Gal4, which is consistent with the observation that *faf* mutant flies do not display any abnormalities in the wing or the notum (Overstreet *et al.* 2004). These proof-of-principle experiments therefore demonstrated that specific adult phenotypes in the wing and notum associated with defective Notch signaling could be efficiently scored in targeted RNAi screens for all DUBs in the Notch signaling cascade.

**Figure 3:**
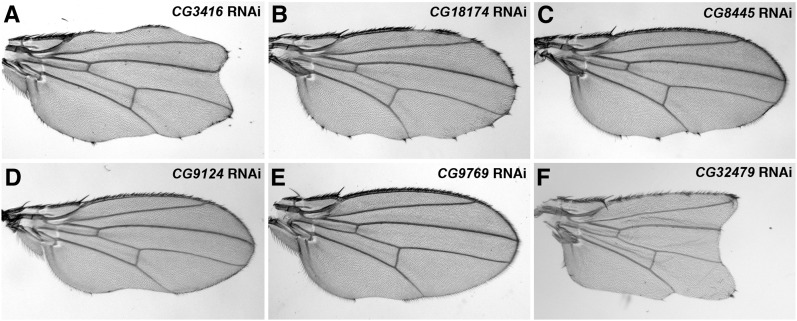
Reduced expression of several DUBs leads to defects on the formation of the margin vein in the adult fly wing. Reduced expression of *CG3416* (A), *CG18174* (B), *CG8445* (C), *CG9124* (D), *CG9769* (E), or *CG32479* (F) in the wing imaginal disc by respective RNAi transgenes driven by the *c96*-Gal4 driver led to defective wing margin formation, with different degrees of severity. Note that the wing margin defects in panels A and F are stronger than those caused by *Notch* RNAi (*cf*. Figure 2D), suggesting that *CG3416*, which encodes an essential component of the 19S proteasome, and probably *CG32479*, play additional roles other than those in Notch signaling. Phenotypes shown in panels A (n = 19), B (n = 9), C (n = 15), D (n = 11), E (n = 6), and F (n = 23) are fully penetrant.

**Figure 4:**
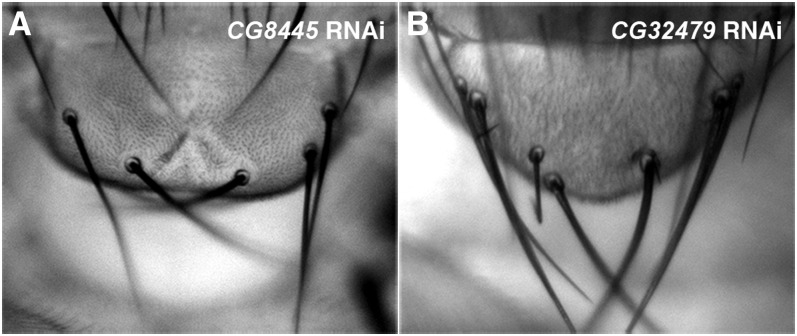
Two candidate DUBs regulate the specification of scutellar bristles in the adult fly notum. Reduced expression of *CG8445* (A) or *CG32479* (B) in the wing imaginal disc by respective RNAi driven by the *dpp*-Gal4 driver resulted in an increased number of scutellar bristles, with a penetrance of 18% (n = 11) for *CG8445* RNAi and 100% (n = 20) for *CG32479* RNAi.

**Figure 5:**
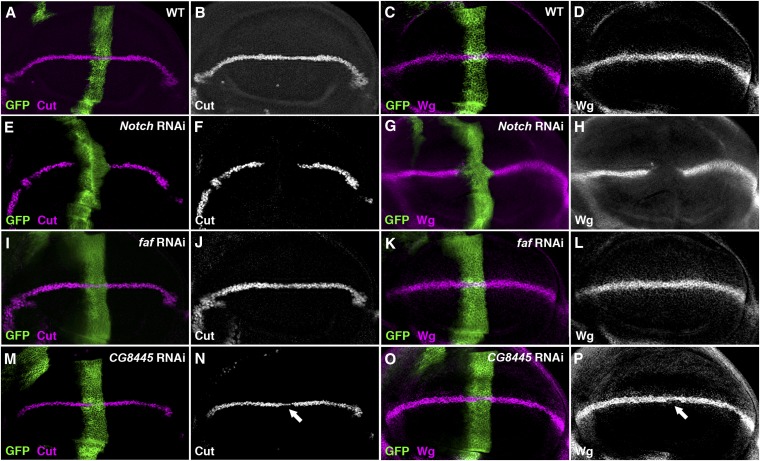
The Effect of *CG8445* RNAi on the expression of Notch signaling targets in the wing imaginal disc. The expression of Wg (magenta; A) and Cut (magenta; C) along the dorsal-ventral boundary (*i.e.*, presumptive wing margin) is activated by Notch signaling in a wild-type (WT) wing disc. Knocking down the expression of *Notch* receptor by the *ptc*-Gal4 driver along the anterior-posterior boundary (marked by GFP) led to a complete loss of downstream targets, Cut (E, F) and Wg (G, H), on the presumptive wing margin cells intersecting the dorsal-ventral boundary. In contrast, the expression of both Cut (J) and Wg (L) was unaffected in wing discs ectopically expressing *faf* RNAi, consistent with previous studies indicating that *faf* mutant flies did not exhibit any defect in the wing. Knockdown of the expression of *CG8445* by RNAi in the wing disc resulted in modulate reduction of Cut (arrow; N) and Wg (arrow; P), suggesting that *CG8445* is necessary to positively regulate Notch signaling. All phenotypes are fully penetrant (n > 20 discs).

We obtained 99 RNAi lines targeting all 45 DUB genes that we annotated in the fly genome (Table S2). We found that reduced expression of six DUB genes by either the *C96*-Gal4 or *dpp*-Gal4 altered adult wing margin morphology and/or the number of scutellar bristles (Figures 3 and 4; Table S2). As several developmental signaling systems, including Notch, Hedgehog, Wnt and growth factor signaling, collaborate to control the wing development (Blair 2007), a secondary screen was performed in the third-instar larval wing imaginal disc, a primordial tissue of the adult wing, to identify DUBs specifically functioning on Notch signaling. The distribution patterns of Notch signaling targets, Cut and Wg (Figure 5, A−D), along the presumptive wing margin, *i.e.* the dorsal-ventral boundary of the wing disc, were examined when candidate DUB RNAi was respectively overexpressed by either the *ptc*-Gal4 or the *dpp*-Gal4 driver (Table S3). Note that both Gal4 drivers confer RNAi expression along the anterior-posterior border, thus allowing us to compare directly expression patterns of Notch target genes in RNAi expressing cells (*i.e.* anteroposterior border cells intersecting the future wing margin) with those in wild-type margin cells in the same disc (see an example of the effect of *Notch* RNAi on the activation of Cut and Wg in Figure 5, E−H). Only those candidate DUB genes, exhibiting significantly altered target expression when knocked down by RNAi, were chosen as potential Notch regulators.

One of these candidate DUBs, *CG3416* (*Mov34*), encodes the *Drosophila* ortholog of vertebrate Rpn8, an essential regulatory subunit of the 19S proteasome (Glickman *et al.* 1998). As proteasome function is important in many cellular processes, it was not surprising that reduced *CG3416* expression by RNAi with either the *ptc*-Gal4 or *dpp*-Gal4 resulted in early larval lethality, precluding a study of its function in Notch signaling in the developing wing disc (Table S3). In addition, the expression pattern of Cut or Wg was not significantly affected in wing discs overexpressing RNAi specific for *CG18174*, another candidate DUB (Table S3), suggesting that the wing margin defects associated with reduced *CG18174* expression may not be a direct consequence of defective Notch signaling.

**Figure 6:**
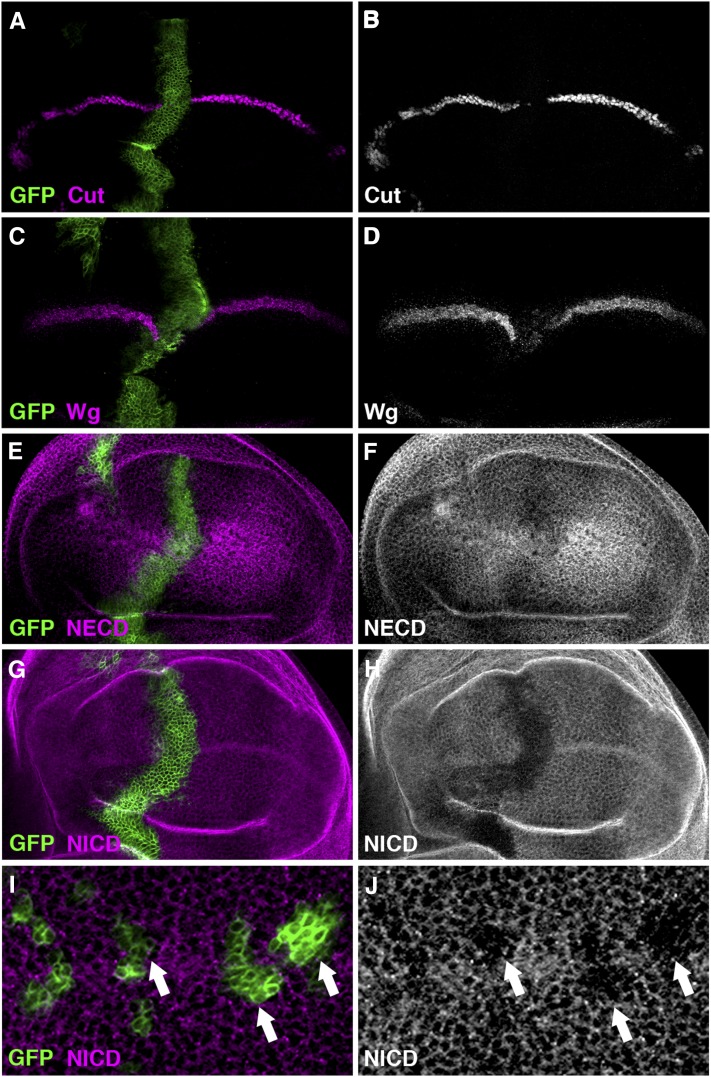
*CG9769* positively regulates Notch signaling in the wing disc. When the expression of *CG9769* was knocked down by RNAi at the anteroposterior boundary of the wing disc by the *dpp*-Gal4 driver (marked by GFP; A and C), Notch signaling activity was largely compromised as indicated by decreased expression of Cut (B) and Wg (D). The abundance of Notch protein, as examined by specific antibodies raised against either the extracellular domain (NECD; E and F) or the intracellular domain (NICD; G and H) of the Notch receptor, also was reduced. The effect of *CG9769* on the production of Notch protein was cell-autonomous (arrows; J), as demonstrated in FLIPout clones (Ito *et al*. 1997) overexpressing *CG9769* RNAi (positively marked by GFP; I). All phenotypes are fully penetrant (n > 20 discs).

**Figure 7:**
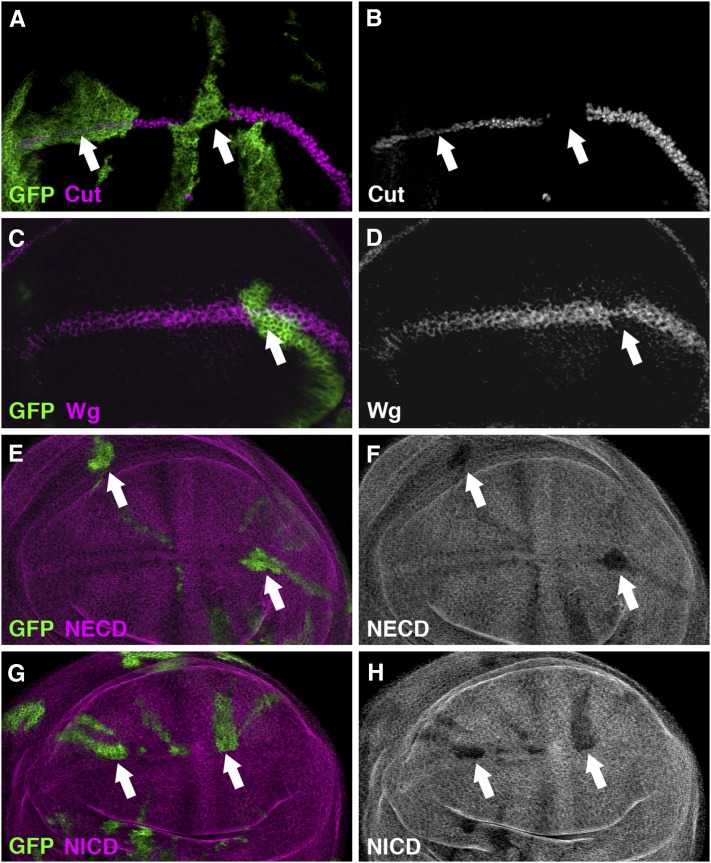
*CG32479* positively regulates Notch signaling in the wing imaginal disc. Random clones overexpressing *CG32479* RNAi were generated by the FLIPout technique. Knocking down the expression of *CG32479* by RNAi in these clones (positively marked by GFP, green) cell-autonomously reduced the expression of Cut (arrows; B) and Wg (arrow; D). Consistently, the production of Notch protein was cell-autonomously downregulated in random clones overexpressing *CG32479* RNAi (positively marked by GFP), as demonstrated by immunostaining with antibodies specific for the NECD (arrows; F) and NICD (arrows; H), respectively. All phenotypes are fully penetrant (n > 20 discs).

In contrast, reducing the expression of each of the remaining four candidate DUBs all led to strong reduction in the expression of Wg and Cut along the future wing margin (Table S3; Figures 5−7), very similar to the effects resulted from reduced *Notch* expression (*cf*. Figure 5, E−H). One of these candidate DUBs, *CG8445* (*calypso*), encodes a *Drosophila* ortholog of vertebrate BRCA1 associated protein 1 (BAP1). Interestingly, a recent study demonstrated that zebrafish BAP1, when knocked down by specific morpholinos, expanded the number of hindbrain neurons in the developing embryo, a neurogenic process dependent on appropriate levels of Notch signaling (Tse *et al.* 2009). Consistent with the function of vertebrate BAP1, we observed increased number of scutellar bristles in the adult fly notum (Figure 4A) and reduced Cut and Wg expression in the wing disc (Figure 5, M−P) when *CG8445* RNAi was overexpressed. These data strongly suggested that CG8455 plays a conserved role like BAP1 to positively regulate Notch signaling.

To our knowledge, the other three DUB genes, *CG9124* (*eIF-3p40*) *CG9769*, and *CG32479*, have not yet been functionally studied *in vivo*. Therefore, we conducted genetic studies to unveil the functions of these genes in the regulation of Notch signaling in *Drosophila*.

### Components of the eIF3 complex function as potential DUBs for the Notch receptor *In Vivo*

Both *CG9124* (*eIF-3p40*) and *CG9769* encode the MJD domain-containing DUB enzymes that are highly conserved from insects to humans (Figure 1). The vertebrate orthologs of protein products of these two DUBs are components of the eukaryotic translation initiation factor 3 (eIF3) complex. CG9769 is the fly ortholog of the eIF3 subunit F (eIF3F) with 45.4% homology, whereas CG9124 shares 50% conservation with the subunit H (eIF3H) (Lasko 2000).

Reduced expression of either *CG9124* or *CG9769* by RNAi in the wing disc driven by the *dpp*-Gal4 or *ptc*-Gal4 resulted in lethality at the pupal stage. However, when the respective RNAi was overexpressed using the *C96*-Gal4 driver, we observed a weak loss-of-function Notch phenotype along the wing margin (Figure 3, D and E). To confirm the requirement of the fly eIF3 proteins in Notch signaling, we investigated the effect of reduced expression of *CG9124* or *CG9769* on the activation of Cut and Wg. As expected, reduced expression of *CG9124* or *CG9769* by RNAi in the wing disc led to abolished Cut and Wg expression along the dorsal-ventral boundary (Figure 6, A−D; Figure S2, A−D), providing *in vivo* evidence to support a role of the eIF3 complex in modulating Notch signaling.

Having confirmed their role in Notch signaling, the next question we asked was at which step along the Notch signaling cascade these two components of the fly eIF3 complex function in the wing disc. The clue to this question came from a recent study in cultured mammalian cells, in which eIF3F functions at the level of activated Notch protein (*i.e.*, NICD) to assure its proper entry to the nucleus (Moretti *et al.* 2010). To test whether this mode of regulation is conserved *in vivo*, we examined the expression pattern of Notch protein in the wing disc when either component was knocked down by RNAi. Because the full-length Notch protein is subject to proteolytic cleavages, two Notch antibodies, C458.2H (Diederich *et al.* 1994) and C17.9C6 (Fehon *et al.* 1990), were used to specifically recognize the resulting extracellular (NECD) and intracellular domains (NICD) of the Notch receptor, respectively. We found that the levels of both NECD and NICD were significantly reduced along the anteroposterior boundary when *CG9124* or *CG9769* RNAi was overexpressed by the *dpp*-Gal4 or *ptc*-Gal4 (Figure 6, E−H; Figure S2, E−F). Further analyses of clones overexpressing *CG9769* RNAi, which were positively marked by GFP (Figure 6I), confirmed that *CG9769* acted cell-autonomously to regulate the abundance of the Notch receptor (Figure 6J). Taken together, our study uncovered an *in vivo* role of components of the eIF3 complex in the positive regulation of the Notch signal transduction, most likely acting at the level of the Notch receptor.

### Identification of CG32479 as a novel DUB that regulates Notch signaling

The last candidate DUB gene *CG32479* encodes the fly ortholog of vertebrate USP10, which contains a carboxy-terminal USP signature domain (Figure 1). Reduced expression of *CG32479* by RNAi resulted in a typical loss-of-function *Notch* phenotype in the adult fly wing and notum: big serrations along the wing margin (Figure 3F) and increased number of scutellar bristles (Figure 4B). Consistent with the adult wing margin phenotype, we found that the expression of Cut (Figure 7B), Wg (Figure 7D) and *Su(H)-lacZ* (Figure S3B) was all downregulated in the wing disc cells overexpressing *CG32479* RNAi. These results, together with the observation that *CG32479* RNAi led to reduced production of Notch proteins (Figure 7, E−H), suggested that *CG32479* is cell-autonomously required in Notch signaling to specify the wing margin.

**Figure 8:**
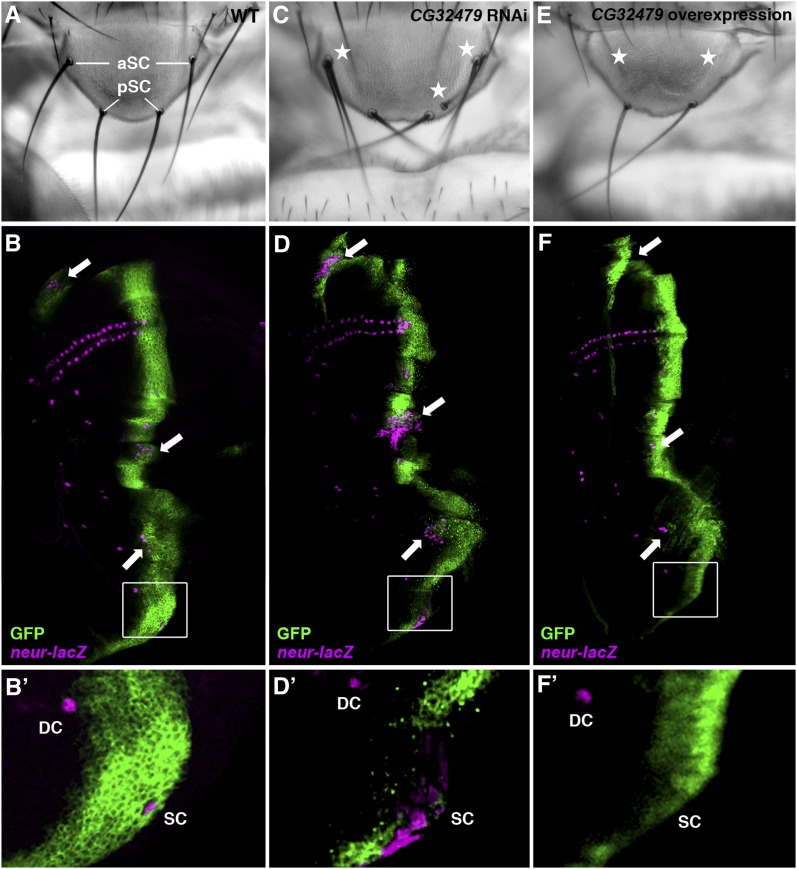
*CG32479* is both necessary and sufficient to control the formation of scutellar bristles. The adult scutellar bristles (A) are differentiated from macrochaete SOP cells in early third-instar wing imaginal discs, which can be marked by the *neur-lacZ* reporter (magenta; B-F′). Knocking down the expression of *CG32479* by the *ptc*-Gal4 driver (green) led to increased numbers of scutellar bristles in the adult notum (stars; C): 64% of flies with 5−6 scutellar bristles and 36% of flies with more than 6 scutellar bristles (n = 83). This fully penetrant scutellar bristle phenotype was a consequence of expanded SOP fate observed in wing discs along the *ptc*-Gal4 expression pattern (arrows; D). In contrast, overexpressing *CG32479* resulted in loss of anterior scutellar bristles (100% penetrant, n = 25; stars; E) and reduction of SOP cells (arrows; F). Enlarged boxed areas shown in B′, D′, and F′ highlight distinct *neur-lacZ* staining in the scutellar (SC) and dorsal-central (DC) SOPs resulted from altered expression of *CG32479*. Note that the expression pattern of the *ptc*-Gal4 driver (marked by GFP; B-F′) overlaps the SC but not the DC SOPs.

To gain further insight on the role of *CG32479* in Notch signaling, we investigated whether *CG32479* activity was sufficient for activating Notch signaling. As the wing margin formation is not a reliable readout for activated Notch signaling, we investigate consequences of manipulated *CG32479* expression on scutellar bristle differentiation. The number of scutellar bristles in the fly notum is determined through a lateral inhibition process in which the fate of sensory organ precursors (SOPs) is specified from the scutellar proneural cluster. It is known that an appropriate Notch signaling activity is required for correct SOP specification. Reduced Notch signaling leads to increased SOP specification to generate extra scutellar bristles in the adult fly notum, whereas heightened Notch activity often results in reduced number of scutellar bristles (de Celis *et al.* 1991; Brennan *et al.* 1999). The development of the adult notum is prepatterned in the wing disc where SOP cells can be visualized by specific markers, including the *neur-lacZ* enhancer trap used in our study [Figure 8B (Huang *et al.* 1991)]. We manipulated the expression of *CG32479* in SOP cells using the *ptc*-Gal4 driver; scutellar SOPs (Figure 8B′) as well as additional SOPs in the wing pouch and other regions of the notum (arrows; Figure 8B) overlap the stripe of *ptc* expression (marked by GFP) in the developing wing disc (Brennan *et al.* 1999). As shown in Figure 8D′, overexpressing *CG32479* RNAi by the *ptc*-Gal4 significantly expanded the number of scutellar (SC) SOP cells as marked by an increased *neur-lacZ* labeling. In addition, we identified two GS (Gene Search) trap fly lines (Toba *et al.* 1999) that can be used to overexpress *CG32479*. Heightened *CG32479* activity conferred by either GS transgene reduced the number of bristles in the scutellum (Figure 8E), a typical phenotype associated with activated Notch signaling. Consistently, we demonstrated that this gain-of-function *Notch* phenotype was a direct consequence of failed scutellar SOP specification (Figure 8F′); the expression of *neuralized-lacZ* was diminished specifically in SC SOPs but not in dorsal-central (DC) SOPs where *CG32479* was not overexpressed. Apart from its effect on scutellar SOPs, altered expression of *CG32479* was sufficient to control SOP specification in other areas of the wing pouch and notum in the wing disc where the *ptc*-Gal4 was expressed (arrows; Figure 8, D and F). Taken together, our results indicated that *CG32479* is not only necessary but also sufficient to positively regulate Notch signaling *in vivo*.

## Discussion

The ubiquitination state of a protein substrate is tightly coordinated by opposing actions of ubiquitination and deubiquitination. Although the ubiquitination process has been extensively studied in the last two decades, the complex roles of deubiquitination have only recently been revealed through the study of a small number of DUBs. Protein deubiquitination is likely to represent an important regulatory mechanism in processes ranging from proteasomal regulation, cell-cycle control, histone modification, membrane trafficking, to stem cell maintenance (Reyes-Turcu *et al.* 2009; Huang *et al.* 2011). Despite progress in uncovering a role for DUBs in several cellular processes, their substrate specificity and modes of regulation in an *in vivo* system are largely unknown. *Drosophila melanogaster* has been widely recognized as an ideal *in vivo* system for understanding complex biological processes due to its genomic conservation with vertebrates and highly amenable genetics. However, only a handful of fly DUBs, including CG1945 (Faf), CG4166 (Not), CG5798 (Ubpy), and CG8445 (Calypso), have been functionally studied in detail (Chen *et al.* 2002; Zhao *et al.* 2008; Mukai *et al.* 2010; Scheuermann *et al.* 2010).

In the current study, we identified and annotated 45 DUBs encoded in the *Drosophila* genome. Like their vertebrate counterparts, the fly DUBs can be categorized into five subfamilies, each with a distinct DUB signature domain. Previous forward genetic screens have yielded several genes that play critical roles in the regulation of the Notch signaling transduction. However, only a single DUB (Faf) was identified in these screens, probably due to the fact that most DUBs are either maternally provided or are pleiotropically required in processes essential for cell survival or differentiation. In contrast, our RNAi-based screen, designed to examine the role of DUBs specifically on the formation of the adult wing and notum, led us to successfully uncover at least four candidate DUBs that potentially regulate Notch signaling. It should be noted that these DUBs were not identified in previous RNAi screens for novel regulators of Notch signaling (Mummery-Widmer *et al.* 2009; Saj *et al.* 2010). One of these screens was based on the usage of the *pnr*-Gal4 driver, which strongly expresses individual RNAi transgenes in the proximal-most part of the presumptive notum in the wing disc (Mummery-Widmer *et al.* 2009). Most of the RNAi transgenes tested in this screen resulted in early lethality or general growth defects in the fly nota, thus making it difficult to identify specific Notch signaling regulators. In our screen, however, we took advantage of the *c96*-Gal4 driver, which confers a much weaker and restricted expression of the RNAi transgenes along the presumptive wing margin. This unique property of the *c96*-Gal4 allowed us to uncover a specific role of DUBs in a complex biological pathway, such as Notch signaling, from their pleiotropic effects in other cellular and developmental processes in *Drosophila*. Our *in vivo* RNAi screen for DUBs in Notch signaling, coupled with morphological and molecular analyses, provided a framework in which specific roles of individual DUBs can be studied in fly development.

At least four potential DUB regulators of Notch signaling were identified in this study. Knocking down either of these DUBs by RNAi not only significantly altered the adult wing margin formation and/or notal bristle formation but also reduced the expression of Notch signaling targets, thus suggesting a positive role of candidate DUBs in Notch signaling. Previous studies suggested that orthologs of these fly DUBs function at the level of transcription or posttranscription in a variety of cellular processes (Bomberger *et al.* 2010; Moretti *et al.* 2010; Scheuermann *et al.* 2010; Yuan *et al.* 2010). It is highly plausible that the fly DUBs confer a multi-level regulation along the Notch signaling cascade.

One of candidate DUBs, CG8445 (Calypso), has been reported to function in the Polycomb repressive deubiquitinase complex (PR-DUB) to remove mono-ubiquitination from histone H2A in nucleosomes. Reduced expression of *CG8445* disrupts the PR-DUB complex, resulting in elevated levels of mono-ubiquitinated H2A to globally de-repress target gene transcription in *Drosophila* (Scheuermann *et al.* 2010). Accordingly, the effect of *CG8445* RNAi observed in our study could be a consequence of epigenetic effects of the PR-DUB on a negative regulator of Notch signaling; increased transcription of this negative regulator downregulates Notch signaling. Alternatively, CG8445 could act independently of the PR-DUB, thereby directly deubiquitinating a key component of the Notch signaling pathway. This notion is supported by the fact that a genome-wide chromatin immunoprecipitation assay failed to detect any significant binding of the PR-DUB on genomic loci of known Notch signaling regulator genes (Scheuermann *et al.* 2010).

Our work on *CG9769* and *CG9124* (*eIF*-*3p40*), which encode the fly orthologs of eIF3F and eIF3H, respectively (Lasko 2000), reveals an *in vivo* function of the eIF3 complex members in Notch signaling. The eIF3 complex serves as a scaffold to facilitate interactions among several other eIF complexes to participate in the different reactions involved in translation (Hinnebusch 2006). eIF3F and eIF3H are two indispensable components among the 13 eIF3 components identified to date (Masutani *et al.* 2007; Zhou *et al.* 2008). Furthermore, reduced expression of fly orthologs of other eIF3 proteins, including eIF3I (CG8882, Trip1) and eIF3L (CG5642), did not result in any adult phenotype associated with Notch signaling (Mummery-Widmer *et al.* 2009; Saj *et al.* 2010). Thus, we believe that the function of CG9124 and CG9769 on Notch signaling may be independent of their role on translation initiation. Indeed, a recent study suggests that eIF3F could act potentially as a JAMM-containing DUB to regulate the translocation of NICD to the nucleus in cultured mammalian cells (Moretti *et al.* 2010). The JAMM domain in eIF3F and eIF3H is largely conserved across different species. CG9124 and CG9769 may function in a similar manner as their vertebrate counterparts on Notch signaling. It is interesting to note that *CG9769* RNAi was able to downregulate both NICD and NECD, raising an intriguing possibility that the fly eIF3F may function at the level of full-length Notch receptor.

The last candidate DUB identified in our screen is CG32479, which is both necessary and sufficient to positively regulate Notch signaling. Our results further suggested that *CG32479* might function at the same level as or upstream of *Notch*. Additional genetic and biochemical studies will be needed to identify its direct substrate protein. The vertebrate ortholog of CG32479 is USP10, which is known to regulate membrane protein trafficking and deubiquitination of tumor suppressor p53 (Faus *et al.* 2005; Bomberger *et al.* 2009, 2010; Yuan *et al.* 2010; Draker *et al.* 2011; Liu *et al.* 2011). As the regulatory functions of two other DUBs identified in our screen on Notch signaling are conserved, it would be interesting to investigate whether USP10 also participates in the regulation of Notch signaling activity in mammals.

Several key components of the Notch signaling cascade are subject to ubiquitination (Lai 2002; LeBras *et al.* 2011; Weinmaster and Fischer 2011). It is believed that the nature of the ubiquitination pattern (mono- v.s. poly-ubiquitination) or specific lysine residues that are modified by ubiquitin on a single substrate protein may result in distinct outcomes in its subcellular localization, stability or signaling activity (Ciechanover 2005; Mukhopadhyay and Riezman 2007; Clague and Urbé 2010; Ikeda *et al.* 2010; Grabbe *et al.* 2011). Thus, multiple DUBs could be employed to target the same substrate protein, such as the Notch receptor, to confer its different activities in the Notch signaling transduction. Moreover, the requirement of Notch signaling and its regulation are heavily context dependent *in vivo*. For example, Faf, the only known DUB for Notch signaling, functions specifically in the developing eye (Fischer-Vize *et al.* 1992; Cadavid *et al.* 2000; Overstreet *et al.* 2004). Similarly, although all four candidate DUBs play a role in the formation of the wing margin and the patterning of the eye (Figure S4, C−F) in our study, only *CG32479* and *CG8445* are required for the specification of scutellar bristles as knocking down the expression of *CG9124* and *CG9769* had no effect on SOP differentiation (Figure S4, I and J).

Finally, it should be pointed out that we could not rule out the possibility that additional DUBs, other than those identified in our screen, may regulate Notch signaling during the development of other organs/tissues. We surveyed the Flybase (http://flybase.org) for potential alleles of the individual DUB genes, and found molecularly defined *P*-element insertions at the loci of 17 fly DUBs. Additional *P*-element insertions also are present less than 1.5 kbp away from the loci of another 27 DUBs; *CG1950* is the only DUB that does not contain ready-to-use genetic resources at or near its locus. Nevertheless, all these genetic resources are potentially useful for generating loss-of-function DUB alleles, which will help obtain a complete profile of specific DUBs in Notch signaling as well as identify their *bona fide in vivo* targets in development.

## Supplementary Material

Supporting Information
