# Supplementary material for: A Targeted In Vivo RNAi Screen Reveals Deubiquitinases as New Regulators of Notch Signaling
Source: G3 (Bethesda). 2012 Dec 1;2(12):1563–75. doi: 10.1534/g3.112.003780 (PMC3516478; doi:10.1534/g3.112.003780)
Supplement: Supporting Information [file supp_2_12_1563__index.html]

Supporting Information 

# A Targeted *In Vivo* RNAi Screen Reveals Deubiquitinases as New Regulators of Notch Signaling

## Supporting Information for Zhang *et al.*, 2012

**Files in this Data Supplement:**

- Supporting Information - Figures S1-S4 and Tables S1-S3 (PDF, 3 MB)
- Figure S1 - Domain Architectures of the Fly CG4166 and Its Yeast and Human Orthologs (PDF, 145 KB)
- Figure S2 - *CG9124* Positively Regulates Notch Signaling in the Wing Disc (PDF, 1 MB)
- Figure S3 - The Effect of Reduced Expression of *CG32479* on the Activity of the *Su(H)-lacZ* Reporter (PDF, 485 KB)
- Figure S4 - The Effects of Candidate DUBs on Eye Development and Scutellar Bristle Differentiation (PDF, 2 MB)
- Table S1 - Vertebrate Orthologs of Annotated *Drosophila* DUBs (PDF, 75 KB)
- Table S2 - The Complete Data of the RNAi-based Screen for Novel DUB Regulators of Notch Signaling in *Drosophila* (PDF, 89 KB)
- Table S3 - Effects of Reduced Expression of Candidate DUBs on Notch Signaling Targets (PDF, 47 KB)
