# Supplementary material for: A Targeted In Vivo RNAi Screen Reveals Deubiquitinases as New Regulators of Notch Signaling
Source: G3 (Bethesda). 2012 Dec 1;2(12):1563–75. doi: 10.1534/g3.112.003780 (PMC3516478; doi:10.1534/g3.112.003780)
Supplement: Supporting Information [file supp_2.12.1563_003780SI.pdf]

Zhang *et al.*, Figure S1

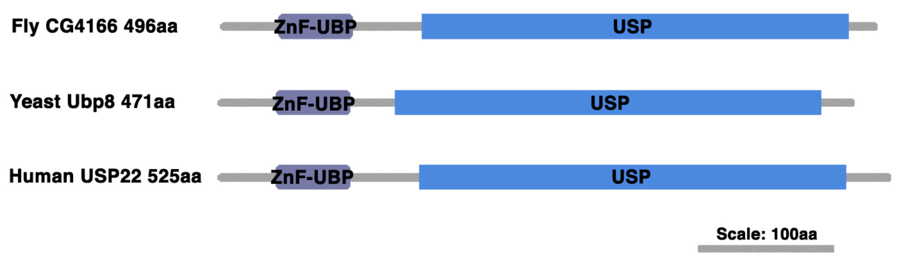

Figure S1 Domain Architectures of the Fly CG4166 and Its Yeast and Human Orthologs. The fly *CG4166*, yeast *ubp8* and vertebrate *usp22* encode orthologous proteins belonging to the USP sub-family of DUBs. All three proteins contain a unique ZnF-UBP domain (zinc-finger ubiquitin binding domain) located N-terminal to the USP signature DUB domain.

Zhang *et al.*, Figure S2

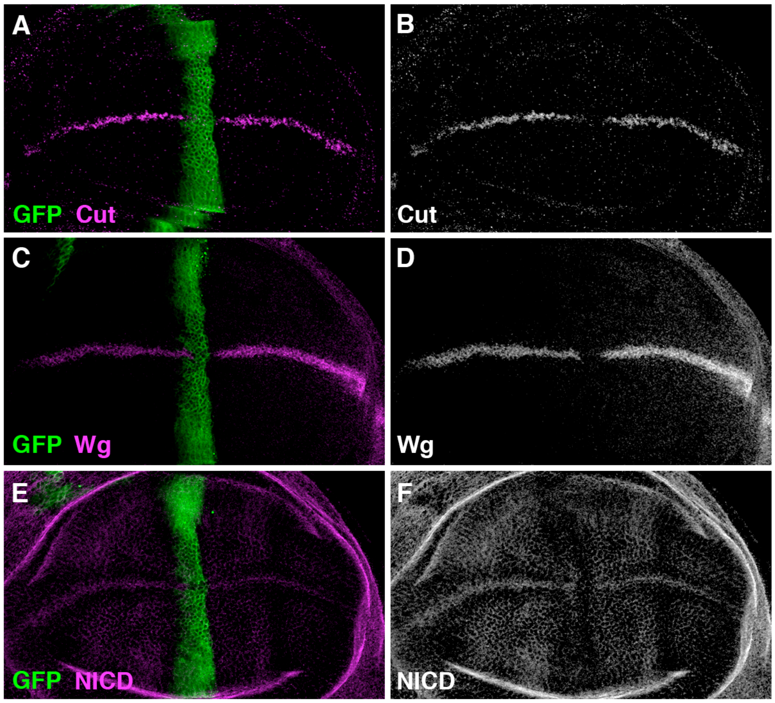

Figure S2 *CG9124* Positively Regulates Notch Signaling in the Wing Disc. When the expression of *CG9124* was knocked down by RNAi along the anterior-posterior boundary using the *ptc*-Gal4 driver (marked by GFP; A, C and E), the activity of Notch signaling and the abundance of Notch protein were reduced as indicated by decreased expression of Cut (B), Wg (D) and NICD (F). All phenotypes are fully penetrant (n>20 discs).

Zhang *et al.*, Figure S3

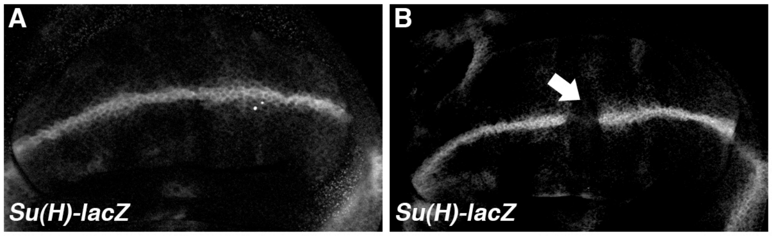

Figure S3 The Effect of Reduced Expression of *CG32479* on the Activity of the *Su(H)-lacZ* Reporter. The activity of one of the downstream targets of Notch signaling, the *Su(H)-lacZ* reporter, was visualized by a b-Galactosidase antibody along the presumptive wing margin in the wing disc (A). When *CG32479* RNAi was overexpressed using the *ptc*-Gal4 driver along the anterior-posterior boundary, the *Su(H)-lacZ* activity was completely abolished in a region of the wing disc where the presumptive wing margin intersects the anterior-posterior boundary (arrow; B). This phenotype is fully penetrant (n=10).

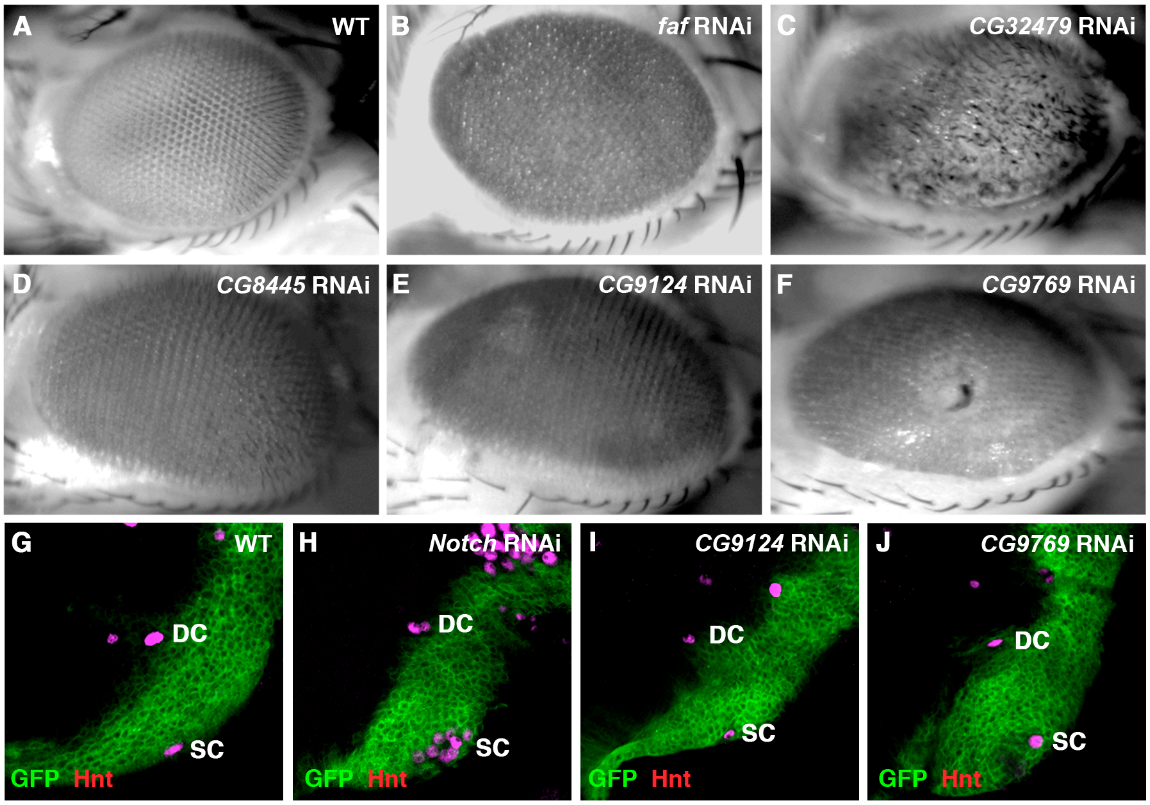

Figure S4 The Effects of Candidate DUBs on Eye Development and Scutellar Bristle Differentiation. Overexpressing *faf* RNAi by the *tub*-Gal4 (B), *CG32479* (C), *CG8445* (D), *CG9124* (E) or *CG9769* (F) RNAi by the *GMR*-Gal4 led to obvious eye patterning defects, as compared with a wildtype adult eye shown in A. During larval development, Hnt (magenta) serves a marker for scutellar (SC) and dorsal-central (DC) SOPs (G) in the wing disc. Reduced *Notch* expression by RNAi using the *dpp*-Gal4 driver significantly expanded the number of SC SOPs in the wing disc (H). Although overexpressing *CG9124* or *CG9769* RNAi led to pupal lethality, the number of Hnt-positive SC SOPs did not change in the larval wing disc. These results suggested that *CG9124* and *CG9769* may not participate in Notch signaling-mediated specification of scutellar bristles. Note that the expression pattern of the *dpp*-Gal4 driver (marked by GFP; G-J) overlaps the SC but not the DC SOPs. All phenotypes are fully penetrant (n>10).

Table S1 Vertebrate Orthologs of Annotated *Drosophila* DUBs. Vertebrate orthologs of the *Drosophila* DUBs were identified based on the NCBI HomoloGene and OrthoDB databases.

Ubiquitin C-terminal Hydrolases; IPR001578: Peptidase C12

Drosophila melanogaster	**Specific DUB Domains**	***Homo sapiens***
CG1950	Peptidase_C12	UCHL5
CG3431 (Uch-L3)	Peptidase_C12	UCHL5
CG4265 (Uch)	Peptidase_C12	UCHL3
CG8445 (Calypso)	Peptidase_C12	BAP1, UCHL2

Ubiquitin-specific Proteases; IPR001394: Peptidase C19

Drosophila melanogaster	**Specific DUB Domains**	***Homo sapiens***
CG12082	Peptidase_C19B	USP5
CG14619	Peptidase_C19R	USP2, USP21
CG1490 (Usp7)	Peptidase_C19C	USP7
CG15817	Peptidase_C19O	USP1
CG1945 (Faf)	Peptidase_C19C	USP9X
CG2904 (Ec)	Peptidase_C19	USP54
CG3016	Peptidase_C19F	USP30
CG30421	Peptidase_C19R	USP31
CG32479	Peptidase_C19	USP10
CG4165	Peptidase_C19K	USP45
CG4166 (Not)	Peptidase_C19D	USP22
CG5384	Peptidase_C19A	USP14
CG5486 (Ubp64E)	Peptidase_C19C	USP47
CG5505 (Scny)	Peptidase_C19E	USP36
CG5603 (CLYD)	Peptidase_C19N	CYLD
CG5794	Peptidase_C19C	USP34
CG5798 (Ubpy)	Peptidase_C19R	UBPY/USP8
CG7023	Peptidase_C19	USP12, USP46
CG7288	Peptidase_C19M	USP39
CG8232	Peptidase_C19	PAN2
CG8334	Peptidase_C19	USP32
CG8494	Peptidase_C19R	USP20, USP33
CG8830	Peptidase_C19H	USP35, USP38

Machado-Joseph Disease Domain Proteases; IPR006155: Machado-Joseph Disease Protein MJD

Drosophila melanogaster	**Specific DUB Domains**	***Homo sapiens***
CG3781	Josephin	JOSD1, JOSD2

Otubain Proteases; IPR003323: Ovarian Tumour, Otubain

Drosophila melanogaster	**Specific DUB Domains**	***Homo sapiens***
CG12743 (Otu)	OTU	
CG3251	OTU	
CG4603	OTU	YOD1
CG4968	OTU	OTUB1
CG6091	OTU	OTUD5
CG7857	OTU	OTUD6B

CG9448 (Trbd)	OTU	ZRANB1

JAMM Domain Proteases; IPR000555: JAB1/Mov34/MPN/PAD-1

Drosophila melanogaster	**Specific DUB Domains**	***Homo sapiens***
CG14884 (CSN5)	MPN_RPN11_CSN5	COPS5
CG18174 (Rpn11)	MPN_RPN11_CSN5	PSMD14
CG2224	MPN_AMSH_like	STAMBP
CG3416 (Mov34)	MPN_RPN7_8	PSMD7
CG4751	MPN_2A_DUB	MPND
CG6932 (CSN6)	MPN_CSN6	COPS6
CG8335 (eIF3-S5)	MPN_eIF3f	EIF3F
CG8877 (Prp8)	MPN_PRP8	PRPF
CG9124 (eIF-3p40)	MPN_eIF3h	EIF3H
CG9769 (eIF3-S5)	MPN_eIF3f	EIF3F

Table S2 The Complete Data of the RNAi-based Screen for Novel DUB Regulators of Notch Signaling in *Drosophila*. Adult wing and scutellar bristle phenotypes were scored when the expression of individual DUBs was knocked down by specific RNAi using the *c96*-Gal4 and *dpp*-Gal4 drivers, respectively.

Ubiquitin C-Terminal Hydrolases, IPR001578, Peptidase C12, Ubiquitin Carboxyl-terminal Hydrolase 1

**CG Number**	**RNAi Strains**	**Wing Phenotypes (*c96*-Gal4)**	**Bristle Phenotypes (*dpp*-Gal4)**	**Other Phenotypes**
CG1950	N1950R-1	No phenotype	No phenotype	a
	N1950R-2	No phenotype	No phenotype	a
CG4265	V103614	No phenotype	No phenotype	a
	N4265R-1	No phenotype	No phenotype	a
	N4265R-2	No phenotype	No phenotype	a
CG3431	V103481	No phenotype	Pupal lethal	f
	N3431R-1	No phenotype	Arrest before 3rd-instar larvae	b
	N3431R-3	No phenotype	Arrest before 3rd-instar larvae	b
CG8445 (calypso)	V107757	Notched wing	More scutellar bristles (SCBs)	f
	N8445R-1	No phenotype	No phenotype [1]	b
	N8445R-2	No phenotype	No phenotype [1]	a
**4 genes**	**11 RNAi strains**			

Ubiquitin-Specific Proteases, IPR001394, Peptidase C19, Ubiquitin Carboxyl-terminal Hydrolase 1

**CG Number**	**RNAi Strains**	**Wing Phenotypes (*c96*-Gal4)**	**Bristle Phenotypes (*dpp*-Gal4)**	**Other Phenotypes**
CG12082	V17567	Loss of margin bristles, wrinkled wing	Pupal lethal	f
	N12082R-2	Loss of margin bristles, wrinkled wing	Less SCBs	b
CG14619	V104382	No phenotype	No phenotype	f
	N14619R-1	No phenotype	No phenotype	b
	N14619R-3	No phenotype	No phenotype	b
CG1490 (Usp-7)	V18231	No phenotype	Less SCBs	c
	V110324	No phenotype	Pupal lethal	f
CG15817	V41604	No phenotype	No phenotype	a
	V100992	No phenotype	No phenotype	f

CG1945 (faf)	V107716	No phenotype	No phenotype	f
	N1945R-1	No phenotype	No phenotype	b
	N1945R-2	No phenotype	No phenotype	b
CG2904 (ec)	V106671	No phenotype	Pupal lethal	f
	N2904R-1	No phenotype	No phenotype	a
	N2904R-2	No phenotype	No phenotype	a
CG3016	V110616	No phenotype	No phenotype	f
	N3016R-1	No phenotype	No phenotype	b
	N3016R-2	No phenotype	No phenotype	b
CG30421	V33726	No phenotype	No phenotype	a
CG32479	V37858	Notched wing, wrinkled wing	Lethal before 3rd-instar larvae [2]	c
	V37859	Notched wing	Pupal lethal	c
CG4165	V110286	No phenotype	No phenotype	f
	N4165R-1	No phenotype	No phenotype	b
	N4165R-2	No phenotype	No phenotype	b
CG4166 (Not)	V45775	Loss of margin bristles, wrinkled wing	Lethal before 3rd-instar larvae [2]	c
	V45776	Loss of margin bristles, wrinkled wing	Lethal before 3rd-instar larvae [2]	c
CG5384	V27405	No phenotype	No phenotype	c
CG5486 (Ubp64E)	V26027	No phenotype	No phenotype	a
	V103743	No phenotype	No phenotype	f
	N5486R-3	No phenotype	No phenotype	a
	N5486R-4	No phenotype	No phenotype	a
CG5505 (scny)	V11152	No phenotype	No phenotype	a
	V105989	No phenotype	Less SCBs	f
CG5603 (CYLD)	V101414	No phenotype	No phenotype	f
	N5603R-1	No phenotype	No phenotype	a
	N5603R-2	No phenotype	No phenotype	a
CG5794	V27517	No phenotype	Pupal lethal [3]	c
	V106192	No phenotype	Pupal lethal [3]	f
CG5798	V107623	Loss of margin bristles, curved wing	Pupal lethal	e
CG7023	V27799	No phenotype	No phenotype	d
CG7288	V47663	Notched wing	Pupal lethal	c

	V110535	Notched wing	Lethal before 3rd-instar larvae	f
	N7288R-1	Notched wing	Pupal lethal [3]	b
	N7288R-2	Loss of margin bristles	Pupal lethal [3]	b
CG8232	N8232R-3	No phenotype	No phenotype	b
CG8334	V18981	No phenotype	No phenotype	a
CG8494	V23934	No phenotype	No phenotype	a
	V28910	No phenotype	No phenotype	a
CG8830	V28960	No phenotype	No phenotype	c
**23 genes**	**49 RNAi strains**			

Machado-Joseph Disease Domain Proteases, IPR006155, Machado-Joseph Disease Protein MJD

CG Number	RNAi Strains	Wing Phenotypes (*c96*-Gal4)	Bristle Phenotypes (*dpp*-Gal4)	Other Phenotypes
CG3781	V7113	No phenotype	No phenotype	a
	V108379	No phenotype	No phenotype	a
**1 gene**	**2 RNAi strains**			

Otubain Proteases, IPR003323, Ovarian Tumour, Otubain

CG Number	RNAi Strains	Wing Phenotypes (*c96*-Gal4)	Bristle Phenotypes (*dpp*-Gal4)	Other Phenotypes
CG12743	V108845	No phenotype	No phenotype	f
	N12743R-1	No phenotype	No phenotype	b
	N12743R-3	No phenotype	No phenotype	b
CG3251	V34573	No phenotype	No phenotype	a
	V100532	No phenotype	Less SCBs	f
CG4603	V21894	No phenotype	No phenotype	a
	N4603R-1	No phenotype	No phenotype	b
	N4603R-2	No phenotype	No phenotype	a
CG4968	V21978	No phenotype	No phenotype	a
CG6091	V27558	No phenotype	No phenotype	c
	V27559	No phenotype	No phenotype	c

	V110659	No phenotype	No phenotype	f
CG7857	V105469	No phenotype	No phenotype	f
	N7857R-2	No phenotype	No phenotype	b
	N7857R-3	No phenotype	No phenotype	b
CG9448	V24030	No phenotype	No phenotype	a
**7 genes**	**16 RNAi strains**			

JAMM Domain Proteases, IPR000555, Mov34/MPN/PAD-1

CG Number	RNAi Strains	Wing Phenotypes (*c96*-Gal4)	Bristle Phenotypes (*dpp*-Gal4)	Other Phenotypes
CG14884	N14884R-1	No phenotype	Reduced scutellum size, 2 shafts in 1 socket	b
CG18174 (Rpn11)	V19272	Notched wing, loss of margin bristles	Pupal lethal [4]	c
	V19273	No phenotype	No phenotype	a
CG2224	V20852	No phenotype	No phenotype	a
	V108622	No phenotype	No phenotype	f
CG3416 (Mov34)	V26183	Notched wing	Pupal lethal	c
	V108573	Notched wing	Pupal lethal	f
CG4751	V26623	No phenotype	No phenotype	a
	V45530	No phenotype	No phenotype	a
CG6932 (CSN6)	V22307	No phenotype	Pupal lethal	c
	V105385	No phenotype	Stunted SCBs, 2 shafts in 1 socket	f
CG8335	V108169	No phenotype	No phenotype	f
	N8335R-1	No phenotype	No phenotype	a
	N8335R-7	No phenotype	No phenotype	a
CG8877 (Prp8)	V18567	Notched wing	Pupal lethal	c
	N8877R-2	Notched wing	Pupal lethal [3]	b
	N8877R-3	Notched wing	Pupal lethal [5]	b
CG9124 (eIF-3p40)	V106189	Loss of margin bristles	Pupal lethal	f
	N9124R-1	No phenotype	No phenotype	b
	N9124R-2	No phenotype	Pupal lethal	b
CG9769 (eIF3-S5)	V101465	Loss of margin bristles	Pupal lethal	f
**10 genes**	**21 RNAi strains**			

[1] Males have extra sex-combs on all legs

[2] Escapers at a lower temperature with more SCBs

[3] Escapers at a lower temperature with less SCBs

[4] Escapers at a lower temperature with reduced scutellum size, less SCBs

[5] Escapers at a lower temperature with less SCBs, 2 shafts in 1 socket

a. No phenotype when overexpressed by different Gal4 drivers in published screens

b. Lethal (semi-lethal) or altered adult structure when driven by the *Act5C*-Gal4 (From the NIG Database)

c. Lethal (semi-lethal) or altered adult structure when driven by the *pnr*-Gal4 (Mummery-Widmer et al. 2009)

d. Lethal when driven by the *elav*-Gal4 (Neely et al. 2010)

e. Lethal (semi-lethal) or altered adult structure when driven by the *sd*-Gal4 (Mukai et al. 2010)

f. Not tested in other RNAi-based screens (the NIG Database; Cronin et al. 2009; Mummery-Widmer et al. 2009; Pospisilik et al. 2009; Neely et al. 2010a; Neely et al. 2010b; Saj et al. 2010; Neumuller et al, 2011; Valakh et al. 2012)

Table S3 Effects of Reduced Expression of Candidate DUBs on Notch Signaling Targets. Altered expression of Notch signaling targets, Cut and Wg, was resulted from the *ptc*-Gal4-driven RNAi-mediated downregulation of candidate DUBs.

CG Number	Cut Expression	Wg Expression
CG3416 (Mov34)	n/e [1]	n/e [1]
CG18174 (Rpn11)	Not obviously changed	Not obviously changed
CG8445 (calypso)	Slightly down	Slightly down
CG9124 (eIF-3p40)	Down	Down
CG9769 (eIF3-S5)	Down	Down
CG32479	Down	Down

[1] Not examined due to early lethality
