# Supplementary material for: A Targeted In Vivo RNAi Screen Reveals Deubiquitinases as New Regulators of Notch Signaling
Source: G3 (Bethesda). 2012 Dec 1;2(12):1563–75. doi: 10.1534/g3.112.003780 (PMC3516478; doi:10.1534/g3.112.003780)
Supplement: Supporting Information [file supp_2.12.1563_FigureS1.pdf]

Zhang *et al.*, Figure S1

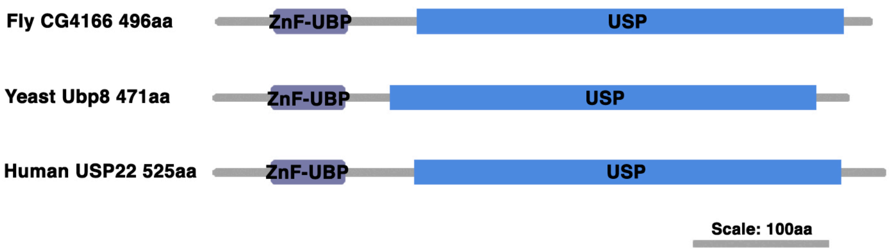

Figure S1 Domain Architectures of the Fly CG4166 and Its Yeast and Human Orthologs. The fly *CG4166*, yeast *ubp8* and vertebrate *usp22* encode orthologous proteins belonging to the USP sub-family of DUBs. All three proteins contain a unique ZnF-UBP domain (zinc-finger ubiquitin binding domain) located N-terminal to the USP signature DUB domain.
