# Supplementary material for: A Targeted In Vivo RNAi Screen Reveals Deubiquitinases as New Regulators of Notch Signaling
Source: G3 (Bethesda). 2012 Dec 1;2(12):1563–75. doi: 10.1534/g3.112.003780 (PMC3516478; doi:10.1534/g3.112.003780)
Supplement: Supporting Information [file supp_2.12.1563_FigureS2.pdf]

Zhang *et al.*, Figure S2

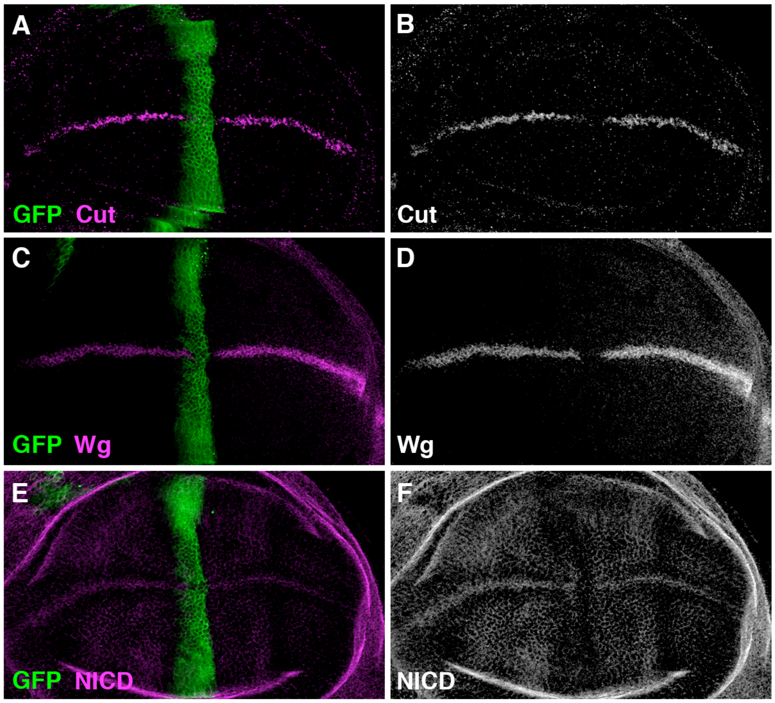

Figure S2 *CG9124* Positively Regulates Notch Signaling in the Wing Disc. When the expression of *CG9124* was knocked down by RNAi along the anterior-posterior boundary using the *ptc*-Gal4 driver (marked by GFP; A, C and E), the activity of Notch signaling and the abundance of Notch protein were reduced as indicated by decreased expression of Cut (B), Wg (D) and NICD (F). All phenotypes are fully penetrant (n>20 discs).
