# Supplementary material for: A Targeted In Vivo RNAi Screen Reveals Deubiquitinases as New Regulators of Notch Signaling
Source: G3 (Bethesda). 2012 Dec 1;2(12):1563–75. doi: 10.1534/g3.112.003780 (PMC3516478; doi:10.1534/g3.112.003780)
Supplement: Supporting Information [file supp_2.12.1563_FigureS3.pdf]

Zhang *et al.*, Figure S3

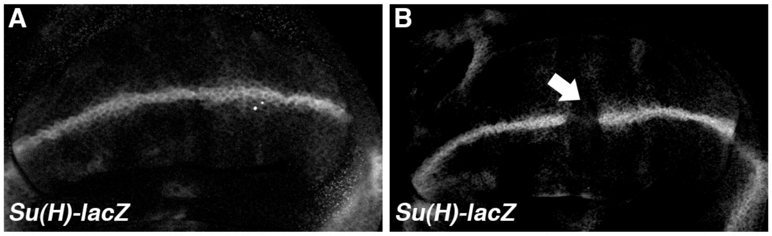


**Figure S3** The Effect of Reduced Expression of *CG32479* on the Activity of the *Su(H)-lacZ* Reporter. The activity of one of the downstream targets of Notch signaling, the *Su(H)-lacZ* reporter, was visualized by a b-Galactosidase antibody along the presumptive wing margin in the wing disc (A). When *CG32479* RNAi was overexpressed using the *ptc*-Gal4 driver along the anterior-posterior boundary, the *Su(H)-lacZ* activity was completely abolished in a region of the wing disc where the presumptive wing margin intersects the anterior-posterior boundary (arrow; B). This phenotype is fully penetrant (n=10).
