# Supplementary material for: A Targeted In Vivo RNAi Screen Reveals Deubiquitinases as New Regulators of Notch Signaling
Source: G3 (Bethesda). 2012 Dec 1;2(12):1563–75. doi: 10.1534/g3.112.003780 (PMC3516478; doi:10.1534/g3.112.003780)
Supplement: Supporting Information [file supp_2.12.1563_FigureS4.pdf]

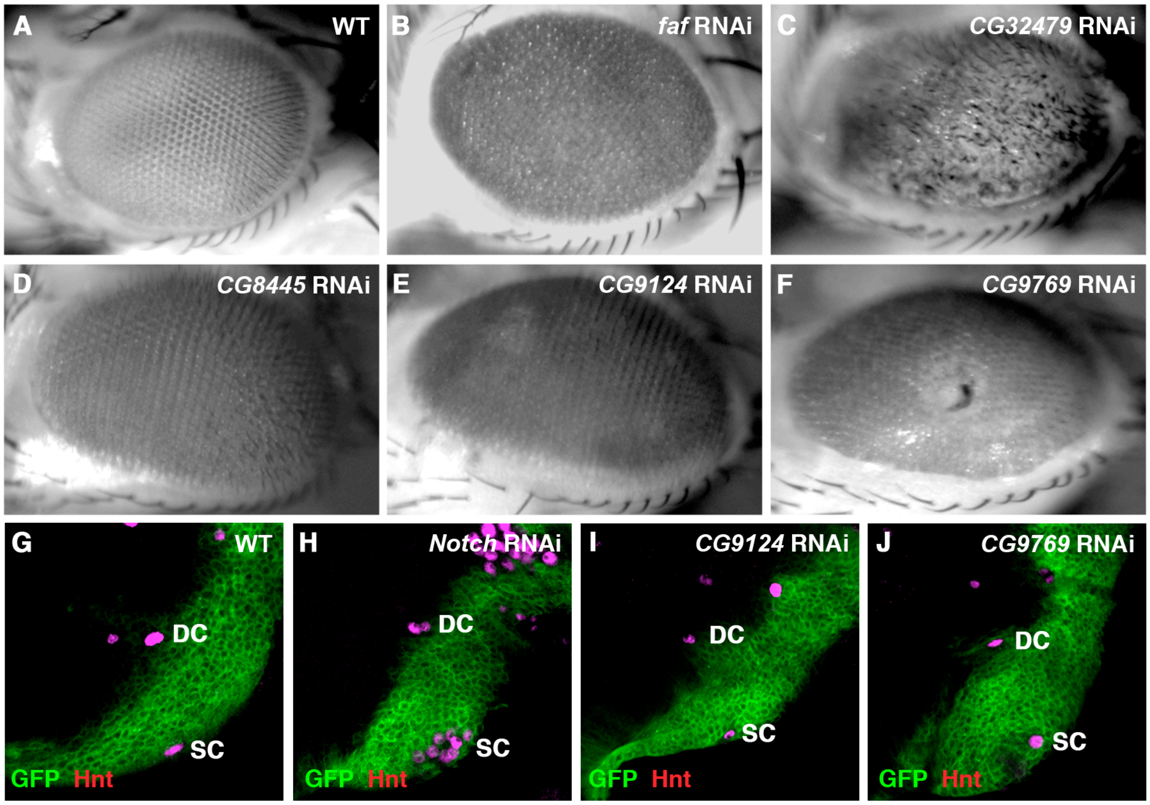

Figure S4 The Effects of Candidate DUBs on Eye Development and Scutellar Bristle Differentiation. Overexpressing *faf* RNAi by the *tub*-Gal4 (B), *CG32479* (C), *CG8445* (D), *CG9124* (E) or *CG9769* (F) RNAi by the *GMR*-Gal4 led to obvious eye patterning defects, as compared with a wildtype adult eye shown in A. During larval development, Hnt (magenta) serves a marker for scutellar (SC) and dorsal-central (DC) SOPs (G) in the wing disc. Reduced *Notch* expression by RNAi using the *dpp*-Gal4 driver significantly expanded the number of SC SOPs in the wing disc (H). Although overexpressing *CG9124* or *CG9769* RNAi led to pupal lethality, the number of Hnt-positive SC SOPs did not change in the larval wing disc. These results suggested that *CG9124* and *CG9769* may not participate in Notch signaling-mediated specification of scutellar bristles. Note that the expression pattern of the *dpp*-Gal4 driver (marked by GFP; G-J) overlaps the SC but not the DC SOPs. All phenotypes are fully penetrant (n>10).
