# Supplementary material for: A Targeted In Vivo RNAi Screen Reveals Deubiquitinases as New Regulators of Notch Signaling
Source: G3 (Bethesda). 2012 Dec 1;2(12):1563–75. doi: 10.1534/g3.112.003780 (PMC3516478; doi:10.1534/g3.112.003780)
Supplement: Supporting Information [file supp_2.12.1563_TableS1.pdf]

Table S1 Vertebrate Orthologs of Annotated *Drosophila* DUBs. Vertebrate orthologs of the *Drosophila* DUBs were identified based on the NCBI HomoloGene and OrthoDB databases.

Ubiquitin C-terminal Hydrolases; IPR001578: Peptidase C12

Drosophila melanogaster	**Specific DUB Domains**	***Homo sapiens***
CG1950	Peptidase_C12	UCHL5
CG3431 (Uch-L3)	Peptidase_C12	UCHL5
CG4265 (Uch)	Peptidase_C12	UCHL3
CG8445 (Calypso)	Peptidase_C12	BAP1, UCHL2

Ubiquitin-specific Proteases; IPR001394: Peptidase C19

Drosophila melanogaster	**Specific DUB Domains**	***Homo sapiens***
CG12082	Peptidase_C19B	USP5
CG14619	Peptidase_C19R	USP2, USP21
CG1490 (Usp7)	Peptidase_C19C	USP7
CG15817	Peptidase_C19O	USP1
CG1945 (Faf)	Peptidase_C19C	USP9X
CG2904 (Ec)	Peptidase_C19	USP54
CG3016	Peptidase_C19F	USP30
CG30421	Peptidase_C19R	USP31
CG32479	Peptidase_C19	USP10
CG4165	Peptidase_C19K	USP45
CG4166 (Not)	Peptidase_C19D	USP22
CG5384	Peptidase_C19A	USP14
CG5486 (Ubp64E)	Peptidase_C19C	USP47
CG5505 (Scny)	Peptidase_C19E	USP36
CG5603 (CLYD)	Peptidase_C19N	CYLD
CG5794	Peptidase_C19C	USP34
CG5798 (Ubpy)	Peptidase_C19R	UBPY/USP8
CG7023	Peptidase_C19	USP12, USP46
CG7288	Peptidase_C19M	USP39
CG8232	Peptidase_C19	PAN2
CG8334	Peptidase_C19	USP32
CG8494	Peptidase_C19R	USP20, USP33
CG8830	Peptidase_C19H	USP35, USP38

Machado-Joseph Disease Domain Proteases; IPR006155: Machado-Joseph Disease Protein MJD

Drosophila melanogaster	**Specific DUB Domains**	***Homo sapiens***
CG3781	Josephin	JOSD1, JOSD2

Otubain Proteases; IPR003323: Ovarian Tumour, Otubain

Drosophila melanogaster	**Specific DUB Domains**	***Homo sapiens***
CG12743 (Otu)	OTU	
CG3251	OTU	
CG4603	OTU	YOD1
CG4968	OTU	OTUB1
CG6091	OTU	OTUD5
CG7857	OTU	OTUD6B

CG9448 (Trbd)	OTU	ZRANB1

JAMM Domain Proteases; IPR000555: JAB1/Mov34/MPN/PAD-1

Drosophila melanogaster	**Specific DUB Domains**	***Homo sapiens***
CG14884 (CSN5)	MPN_RPN11_CSN5	COPS5
CG18174 (Rpn11)	MPN_RPN11_CSN5	PSMD14
CG2224	MPN_AMSH_like	STAMBP
CG3416 (Mov34)	MPN_RPN7_8	PSMD7
CG4751	MPN_2A_DUB	MPND
CG6932 (CSN6)	MPN_CSN6	COPS6
CG8335 (eIF3-S5)	MPN_eIF3f	EIF3F
CG8877 (Prp8)	MPN_PRP8	PRPF
CG9124 (eIF-3p40)	MPN_eIF3h	EIF3H
CG9769 (eIF3-S5)	MPN_eIF3f	EIF3F
