# Supplementary material for: A Targeted In Vivo RNAi Screen Reveals Deubiquitinases as New Regulators of Notch Signaling
Source: G3 (Bethesda). 2012 Dec 1;2(12):1563–75. doi: 10.1534/g3.112.003780 (PMC3516478; doi:10.1534/g3.112.003780)
Supplement: Supporting Information [file supp_2.12.1563_TableS2.pdf]

Table S2 The Complete Data of the RNAi-based Screen for Novel DUB Regulators of Notch Signaling in *Drosophila.* Adult wing and scutellar bristle phenotypes were scored when the expression of individual DUBs was knocked down by specific RNAi using the *c96*-Gal4 and *dpp*-Gal4 drivers, respectively.

Ubiquitin C-Terminal Hydrolases, IPR001578, Peptidase C12, Ubiquitin Carboxyl-terminal Hydrolase 1

**CG Number**	**RNAi Strains**	**Wing Phenotypes (*c96*-Gal4)**	**Bristle Phenotypes (*dpp*-Gal4)**	**Other Phenotypes**
CG1950	N1950R-1	No phenotype	No phenotype	a
	N1950R-2	No phenotype	No phenotype	a
CG4265	V103614	No phenotype	No phenotype	a
	N4265R-1	No phenotype	No phenotype	a
	N4265R-2	No phenotype	No phenotype	a
CG3431	V103481	No phenotype	Pupal lethal	f
	N3431R-1	No phenotype	Arrest before 3rd-instar larvae	b
	N3431R-3	No phenotype	Arrest before 3rd-instar larvae	b
CG8445 (calypso)	V107757	Notched wing	More scutellar bristles (SCBs)	f
	N8445R-1	No phenotype	No phenotype [1]	b
	N8445R-2	No phenotype	No phenotype [1]	a
**4 genes**	**11 RNAi strains**			

Ubiquitin-Specific Proteases, IPR001394, Peptidase C19, Ubiquitin Carboxyl-terminal Hydrolase 1

**CG Number**	**RNAi Strains**	**Wing Phenotypes (*c96*-Gal4)**	**Bristle Phenotypes (*dpp*-Gal4)**	**Other Phenotypes**
CG12082	V17567	Loss of margin bristles, wrinkled wing	Pupal lethal	f
	N12082R-2	Loss of margin bristles, wrinkled wing	Less SCBs	b
CG14619	V104382	No phenotype	No phenotype	f
	N14619R-1	No phenotype	No phenotype	b
	N14619R-3	No phenotype	No phenotype	b
CG1490 (Usp-7)	V18231	No phenotype	Less SCBs	c
	V110324	No phenotype	Pupal lethal	f
CG15817	V41604	No phenotype	No phenotype	a
	V100992	No phenotype	No phenotype	f

CG1945 (faf)	V107716	No phenotype	No phenotype	f
	N1945R-1	No phenotype	No phenotype	b
	N1945R-2	No phenotype	No phenotype	b
CG2904 (ec)	V106671	No phenotype	Pupal lethal	f
	N2904R-1	No phenotype	No phenotype	a
	N2904R-2	No phenotype	No phenotype	a
CG3016	V110616	No phenotype	No phenotype	f
	N3016R-1	No phenotype	No phenotype	b
	N3016R-2	No phenotype	No phenotype	b
CG30421	V33726	No phenotype	No phenotype	a
CG32479	V37858	Notched wing, wrinkled wing	Lethal before 3rd-instar larvae [2]	c
	V37859	Notched wing	Pupal lethal	c
CG4165	V110286	No phenotype	No phenotype	f
	N4165R-1	No phenotype	No phenotype	b
	N4165R-2	No phenotype	No phenotype	b
CG4166 (Not)	V45775	Loss of margin bristles, wrinkled wing	Lethal before 3rd-instar larvae [2]	c
	V45776	Loss of margin bristles, wrinkled wing	Lethal before 3rd-instar larvae [2]	c
CG5384	V27405	No phenotype	No phenotype	c
CG5486 (Ubp64E)	V26027	No phenotype	No phenotype	a
	V103743	No phenotype	No phenotype	f
	N5486R-3	No phenotype	No phenotype	a
	N5486R-4	No phenotype	No phenotype	a
CG5505 (scny)	V11152	No phenotype	No phenotype	a
	V105989	No phenotype	Less SCBs	f
CG5603 (CYLD)	V101414	No phenotype	No phenotype	f
	N5603R-1	No phenotype	No phenotype	a
	N5603R-2	No phenotype	No phenotype	a
CG5794	V27517	No phenotype	Pupal lethal [3]	c
	V106192	No phenotype	Pupal lethal [3]	f
CG5798	V107623	Loss of margin bristles, curved wing	Pupal lethal	e
CG7023	V27799	No phenotype	No phenotype	d
CG7288	V47663	Notched wing	Pupal lethal	c

	V110535	Notched wing	Lethal before 3rd-instar larvae	f
	N7288R-1	Notched wing	Pupal lethal [3]	b
	N7288R-2	Loss of margin bristles	Pupal lethal [3]	b
CG8232	N8232R-3	No phenotype	No phenotype	b
CG8334	V18981	No phenotype	No phenotype	a
CG8494	V23934	No phenotype	No phenotype	a
	V28910	No phenotype	No phenotype	a
CG8830	V28960	No phenotype	No phenotype	c
**23 genes**	**49 RNAi strains**			

Machado-Joseph Disease Domain Proteases, IPR006155, Machado-Joseph Disease Protein MJD

CG Number	RNAi Strains	Wing Phenotypes (*c96*-Gal4)	Bristle Phenotypes (*dpp*-Gal4)	Other Phenotypes
CG3781	V7113	No phenotype	No phenotype	a
	V108379	No phenotype	No phenotype	a
**1 gene**	**2 RNAi strains**			

Otubain Proteases, IPR003323, Ovarian Tumour, Otubain

CG Number	RNAi Strains	Wing Phenotypes (*c96*-Gal4)	Bristle Phenotypes (*dpp*-Gal4)	Other Phenotypes
CG12743	V108845	No phenotype	No phenotype	f
	N12743R-1	No phenotype	No phenotype	b
	N12743R-3	No phenotype	No phenotype	b
CG3251	V34573	No phenotype	No phenotype	a
	V100532	No phenotype	Less SCBs	f
CG4603	V21894	No phenotype	No phenotype	a
	N4603R-1	No phenotype	No phenotype	b
	N4603R-2	No phenotype	No phenotype	a
CG4968	V21978	No phenotype	No phenotype	a
CG6091	V27558	No phenotype	No phenotype	c
	V27559	No phenotype	No phenotype	c

	V110659	No phenotype	No phenotype	f
CG7857	V105469	No phenotype	No phenotype	f
	N7857R-2	No phenotype	No phenotype	b
	N7857R-3	No phenotype	No phenotype	b
CG9448	V24030	No phenotype	No phenotype	a
**7 genes**	**16 RNAi strains**			

JAMM Domain Proteases, IPR000555, Mov34/MPN/PAD-1

CG Number	RNAi Strains	Wing Phenotypes (*c96*-Gal4)	Bristle Phenotypes (*dpp*-Gal4)	Other Phenotypes
CG14884	N14884R-1	No phenotype	Reduced scutellum size, 2 shafts in 1 socket	b
CG18174 (Rpn11)	V19272	Notched wing, loss of margin bristles	Pupal lethal [4]	c
	V19273	No phenotype	No phenotype	a
CG2224	V20852	No phenotype	No phenotype	a
	V108622	No phenotype	No phenotype	f
CG3416 (Mov34)	V26183	Notched wing	Pupal lethal	c
	V108573	Notched wing	Pupal lethal	f
CG4751	V26623	No phenotype	No phenotype	a
	V45530	No phenotype	No phenotype	a
CG6932 (CSN6)	V22307	No phenotype	Pupal lethal	c
	V105385	No phenotype	Stunted SCBs, 2 shafts in 1 socket	f
CG8335	V108169	No phenotype	No phenotype	f
	N8335R-1	No phenotype	No phenotype	a
	N8335R-7	No phenotype	No phenotype	a
CG8877 (Prp8)	V18567	Notched wing	Pupal lethal	c
	N8877R-2	Notched wing	Pupal lethal [3]	b
	N8877R-3	Notched wing	Pupal lethal [5]	b
CG9124 (eIF-3p40)	V106189	Loss of margin bristles	Pupal lethal	f
	N9124R-1	No phenotype	No phenotype	b
	N9124R-2	No phenotype	Pupal lethal	b
CG9769 (eIF3-S5)	V101465	Loss of margin bristles	Pupal lethal	f
**10 genes**	**21 RNAi strains**			

[1] Males have extra sex-combs on all legs

[2] Escapers at a lower temperature with more SCBs

[3] Escapers at a lower temperature with less SCBs

[4] Escapers at a lower temperature with reduced scutellum size, less SCBs

[5] Escapers at a lower temperature with less SCBs, 2 shafts in 1 socket

a. No phenotype when overexpressed by different Gal4 drivers in published screens

b. Lethal (semi-lethal) or altered adult structure when driven by the *Act5C*-Gal4 (From the NIG Database)

c. Lethal (semi-lethal) or altered adult structure when driven by the *pnr*-Gal4 (Mummery-Widmer et al. 2009)

d. Lethal when driven by the *elav*-Gal4 (Neely et al. 2010)

e. Lethal (semi-lethal) or altered adult structure when driven by the *sd*-Gal4 (Mukai et al. 2010)

f. Not tested in other RNAi-based screens (the NIG Database; Cronin et al. 2009; Mummery-Widmer et al. 2009; Pospisilik et al. 2009; Neely et al. 2010a; Neely et al. 2010b; Saj et al. 2010; Neumuller et al, 2011; Valakh et al. 2012)
