# Supplementary material for: A Targeted In Vivo RNAi Screen Reveals Deubiquitinases as New Regulators of Notch Signaling
Source: G3 (Bethesda). 2012 Dec 1;2(12):1563–75. doi: 10.1534/g3.112.003780 (PMC3516478; doi:10.1534/g3.112.003780)
Supplement: Supporting Information [file supp_2.12.1563_TableS3.pdf]

Table S3 Effects of Reduced Expression of Candidate DUBs on Notch Signaling Targets. Altered expression of Notch signaling targets, Cut and Wg, was resulted from the *ptc*-Gal4-driven RNAi-mediated downregulation of candidate DUBs.

CG Number	Cut Expression	Wg Expression
CG3416 (Mov34)	n/e [1]	n/e [1]
CG18174 (Rpn11)	Not obviously changed	Not obviously changed
CG8445 (calypso)	Slightly down	Slightly down
CG9124 (eIF-3p40)	Down	Down
CG9769 (eIF3-S5)	Down	Down
CG32479	Down	Down

[1] Not examined due to early lethality
